# Naturally occurring deamidated triosephosphate isomerase is a promising target for cell-selective therapy in cancer

**DOI:** 10.1038/s41598-022-08051-0

**Published:** 2022-03-07

**Authors:** Sergio Enríquez-Flores, Luis A. Flores-López, Ignacio De la Mora-De la Mora, Itzhel García-Torres, Isabel Gracia-Mora, Pedro Gutiérrez-Castrellón, Cynthia Fernández-Lainez, Yoalli Martínez-Pérez, Alberto Olaya-Vargas, Paul de Vos, Gabriel López-Velázquez

**Affiliations:** 1 Laboratorio de Biomoléculas y Salud Infantil, Instituto Nacional de Pediatría, Mexico City, Mexico; 2 Laboratorio de Biomoléculas y Salud Infantil, CONACYT-Instituto Nacional de Pediatría, Mexico City, Mexico; 3 Directora de la Unidad de Investigación Preclínica, Facultad de Química, Universidad Nacional Autónoma de México, Mexico City, Mexico; 4 Hospital General Dr. Manuel Gea González, Mexico City, Mexico; 5 Laboratorio de Errores Innatos del Metabolismo y Tamiz, Instituto Nacional de Pediatría, Mexico City, Mexico; 6 Stem Cell Transplantation and Cellular Therapy, Instituto Nacional de Pediatría, Mexico City, Mexico; 7 Department of Pathology and Medical Biology, University of Groningen, University Medical Center Groningen, Groningen, 9713 GZ The Netherlands; 8 Posgrado en Ciencias Biológicas, Universidad Nacional Autónoma de México, Mexico City, Mexico

**Keywords:** Breast cancer, Cancer therapy, Enzymes, Biochemistry, Cancer, Drug discovery, Target identification

## Abstract

Human triosephosphate isomerase (HsTIM) is a central glycolytic enzyme and is overexpressed in cancer cells with accelerated glycolysis. Triple-negative breast cancer is highly dependent on glycolysis and is typically treated with a combination of surgery, radiation therapy, and chemotherapy. Deamidated HsTIM was recently proposed as a druggable target. Although thiol-reactive drugs affect cell growth in deamidated HsTIM-complemented cells, the role of this protein as a selective target has not been demonstrated. To delve into the usefulness of deamidated HsTIM as a selective target, we assessed its natural accumulation in breast cancer cells. We found that deamidated HsTIM accumulates in breast cancer cells but not in noncancerous cells. The cancer cells are selectively programmed to undergo cell death with thiol-reactive drugs that induced the production of methylglyoxal (MGO) and advanced glycation-end products (AGEs). In vivo, a thiol-reactive drug effectively inhibits the growth of xenograft tumors with an underlying mechanism involving deamidated HsTIM. Our findings demonstrate the usefulness of deamidated HsTIM as target to develop new therapeutic strategies for the treatment of cancers and other pathologies in which this post translationally modified protein accumulates.

## Introduction

Since cancer cells show a high capacity for proliferation, they are energy demanding and thus highly dependent on glycolysis^1–3^. Triosephosphate isomerase (TIM) plays a central role in the pay-off phase of glycolysis by isomerizing dihydroxyacetone phosphate (DHAP) to D-glyceraldehyde-3-phosphate (GAP).

Due to the increased glycolysis and upregulation of the related enzymes in cancer cells^4–6^, substrate analogs for glycolytic enzymes have been proposed as anticarcinogenic agents^7–9^. Additionally, regions distinct from catalytic sites are promising for seeking new antitumoral strategies. These findings support the use of deamidated human TIM (HsTIM) as a druggable target^10^.

Deamidation is the apotheosis of the time-related chemical instability inherent to amino acids like asparagine (Asn) and glutamine (Gln) in proteins. In eukaryotes, deamidation is a spontaneous modification of proteins that has potent repercussions on their activity and stability, both in vivo and in vitro^11–13^. Asparagine deamidation into aspartic acid (Asp) and isoAsp results in an irreversible edition of the information that initially was genetically encoded and translated into proteins at the time of their synthesis, altering the primary structure of the protein, introducing a negative charge, and often modifying their secondary and tertiary structures.

Two deamidation sites in HsTIM at positions 16 and 72 have been found, but the deamidation of Asn 16 induces major functional and structural alterations^12^. Continuous catalytic cycles highly promote the deamidation of HsTIM^14^, which is boosted in aging and pathologies such as cancer^15^. The structural characteristics of deamidated HsTIM in transformed bacteria make it prone to selective inhibition with thiol-reactive compounds^10^.

Since glycolysis is not adequately regulated in cancer cells, we hypothesized that deamidated HsTIM commonly accumulates in these cells, enabling its use as an efficient druggable target to selectively exert cell death. Here, the presence and accumulation of deamidated HsTIM were demonstrated in triple-negative breast cancer cells, which showed high sensitivity to the thiol-reactive drugs rabeprazole and auranofin. Both drugs inhibited cellular HsTIM enzyme activity and induced selective cell death.

Nude mice with implanted breast cancer cells treated with rabeprazole exhibited significantly reduced tumor size compared with the tumor size in untreated mice. Additionally, pretreatment of cancer cells with rabeprazole did not lead to tumor formation after implantation in mice.

The central finding of our study is that deamidated HsTIM is naturally present and accumulates in cancer cells but not in noncancer cells. Overall, the results of in vitro (recombinant protein), in situ (cell culture) and in vivo (xenograft murine model) experiments demonstrate that deamidated HsTIM is an efficient druggable target.

## Results

### Deamidated HsTIM is highly inactivated and structurally affected by the drugs rabeprazole and auranofin through Cys modification

**Figure 1:**
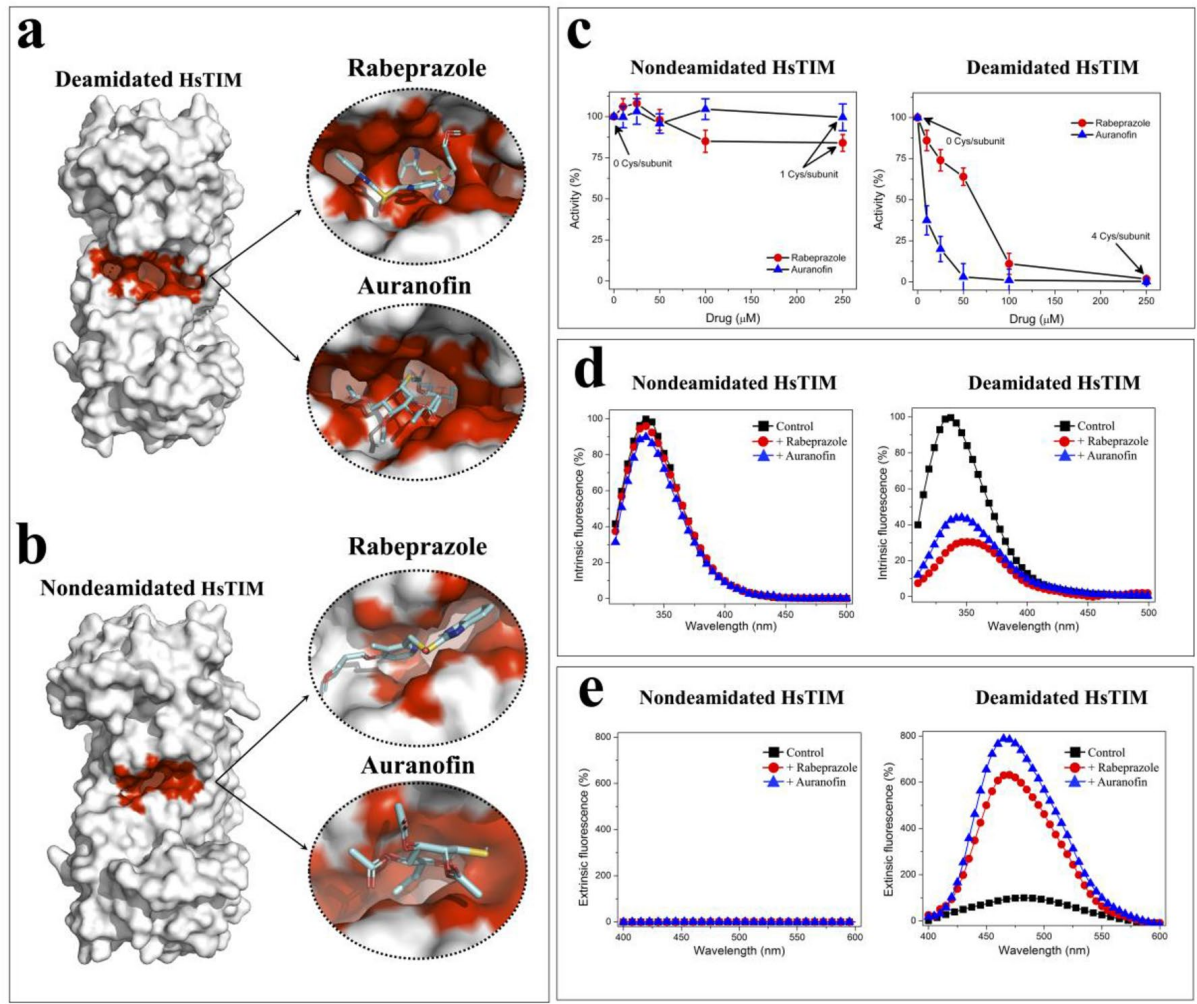
In silico and in vitro studies to analyze the effect of cysteine-reactive drugs rabeprazole and auranofin on HsTIM. Ligand docking in deamidated (**a**) and nondeamidated (**b**) crystallographic structures. (**c)** Inactivation assays with recombinant nondeamidated and deamidated HsTIM proteins. The enzymes were incubated for 2 h at 37 °C with rabeprazole (filled red circle) or auranofin (filled blue triangle) with increasing concentrations from 0 to 250 µM. The number of derivatized Cys is indicated in the absence and in the highest drug concentration. The enzyme activity without the drug was set as 100%. (**d**) Intrinsic fluorescence of nondeamidated and deamidated HsTIM with or without drug treatment. Enzymes were incubated for 2 h at 37 °C in the absence (filled black square) (control) or presence of 250 µM rabeprazole (filled red circle) or 250 µM auranofin (filled blue triangle). The results are expressed as the percent fluorescence, with the maximum fluorescence emission of the control enzyme set at 100%. (**e**) Extrinsic fluorescence of ANS corresponding to the enzymes with or without drug treatment. Enzymes were incubated as above mentioned. The results are expressed as the percent of fluorescence intensity, with the maximum fluorescence emission of the control in deamidated HsTIM set at 100%. The results represent the mean of four independent experiments.

HsTIM is a homodimeric enzyme for which deamidation occurs at amino acid residues located in the contact site between its two subunits (interface), increasing its permeability to small ligands^10,12^. Based on this information, we performed in silico and in vitro analyses of crystallographic structures of nondeamidated and deamidated HsTIM and the corresponding recombinant proteins. The largest and deepest cavity at the interface was found in deamidated HsTIM. Rabeprazole and auranofin were docked at the interface of the crystallographic structure and identified more binding sites in the innermost part of the interface of deamidated HsTIM (Fig. 1a) than in nondeamidated HsTIM (Fig. 1b).

Consistent with the docking data, the enzyme activity of the deamidated HsTIM was completely inhibited, it is, this deamidated protein was fourfold more sensitive to rabeprazole and 30-fold more sensitive to auranofin than nondeamidated HsTIM (Fig. 1c).

The main mechanism of action of both drugs (rabeprazole and auranofin) is covalent binding to the thiolate moieties of cysteine residues (Cys) (named Cys derivatization)^16,17^. The primary structure of HsTIM contains 5 Cys residues per subunit, which were suitably quantified in the recombinant enzyme without drug treatment. Treatment with either rabeprazole or auranofin derivatized 4 Cys residues per subunit in deamidated HsTIM, whereas nondeamidated HsTIM contained only one derivatized Cys residue per subunit after drug treatment (Fig. 1c, and Suppl. Table S1).

The fluorescence spectra of nondeamidated HsTIM showed marginal changes. It means that the tertiary structure did not change upon treatment with either rabeprazole or auranofin (Fig. 1d). In contrast, the intrinsic fluorescence spectra of deamidated HsTIM upon treatment with both drugs was quenched by more than 50% (Fig. 1d). Under these conditions, rabeprazole and auranofin generated 70% and 56% quenching in fluorescence intensity and shifts in the maximum fluorescence emission (λ_max_) of 12 and 9.5 nm, respectively. A shift of λ_max_ toward a longer wavelength (redshift) indicates exposure to the milieu of tryptophan residues previously buried into a folded structure.

To reinforce the structural changes described above, extrinsic fluorescence studies with 8-anilinonaphthalene-1-sulfonic acid (ANS) were performed. ANS is a fluorescent molecular probe that has a high affinity for hydrophobic regions; thus, proteins containing more hydrophobic cavities will exhibit higher ANS fluorescence signals.

The ANS fluorescence in the untreated and treated nondeamidated HsTIM showed a marginal signal; whereas, the fluorescence spectra from ANS in deamidated HsTIM were strongly increased under all conditions (Fig. 1e). This signal was augmented with respect to that of the untreated enzyme by 6.5- and 8-fold upon treatment with rabeprazole and auranofin, respectively. Additionally, the λ_max_ shifted by 14 nm to a lower wavelength (shift to blue) with both drugs. These results show that as a consequence of the drug treatments, deamidated HsTIM became highly permeable to small molecules such as ANS.

### Deamidated HsTIM is present and accumulates in breast cancer cells

To look for the presence of deamidated HsTIM in breast cancer cells, a previously validated method of selective cleavage by hydroxylamine was used^18,19^. Hydroxylamine can selectively cleave the intermediary succinimide that is formed by the side chains of Asn and glycine (Gly) in nondeamidated proteins under alkaline conditions; furthermore, in deamidated proteins, a chain composed of Asp-Gly (or isoAsp-Gly) does not form succinimide under the same conditions; therefore, a deamidated peptide is not cleaved^20^. Then, as HsTIM contains two Asn-Gly pairs at positions 16–17 and 72–73, cleavage might generate three peptides that are 1.87, 5.96 and 18.87 kDa in size if the enzyme is not deamidated. When the cleavage of nondeamidated HsTIM is incomplete, a peptide with a size of 7.83 kDa could also be produced. On the other hand, the cleavage of deamidated HsTIM at only position 16 would produce peptides with sizes of 7.83 and 18.87 kDa, whereas twice deamidated HsTIM would not be cleaved at position 16 or 72. Based on the above, the cleavage patterns of the recombinant enzymes *versus* cellular HsTIM from human primary mammary epithelial cells (HMECs) (normal breast cells) and MDA-MB-231 cells (triple-negative breast cancer cells) were compared.

**Figure 2:**
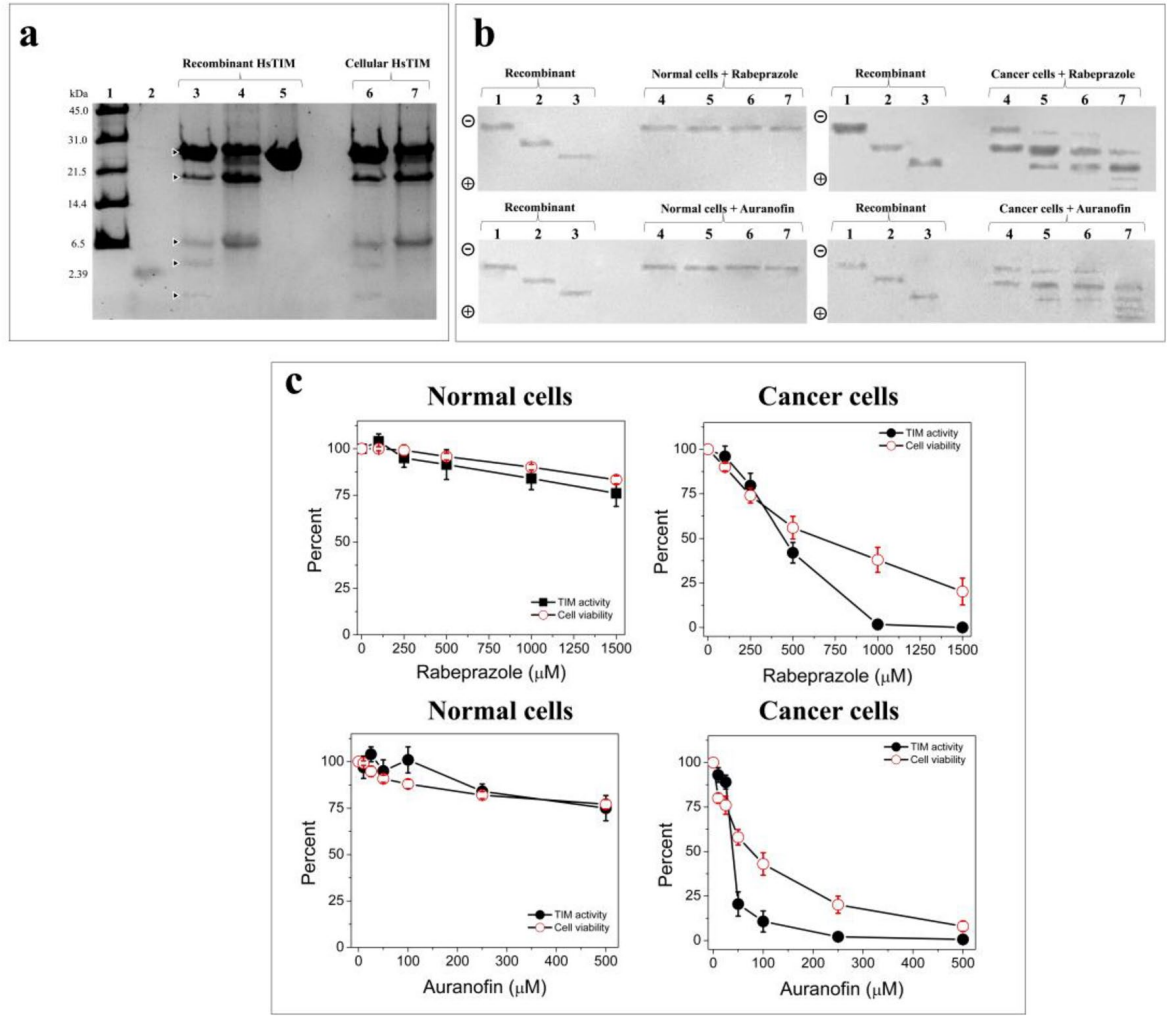
Accumulation of deamidated HsTIM in breast cancer cells and the effects exerted by treatment with thiol drugs. (**a**) SDS-PAGE of hydroxylamine cleavage of the recombinant and cellular HsTIMs: Lane 1: molecular weight standard, lane 2: peptide with a size of 2.39 kDa, lanes 3 to 5: (10 µg of protein/lane) nondeamidated, once deamidated, and twice deamidated HsTIMs. Lanes 6 and 7: (20 µg of protein/lane) immunoprecipitated HsTIM from normal and cancer cells, respectively. (**b**) Western blot analysis of recombinant and cellular HsTIMs in nPAGE: Lanes 1 to 3 (1 µg of protein/lane), recombinant HsTIMs nondeamidated, once deamidated and twice deamidated, respectively. Lanes 4 to 7 (100 µg protein/lane), total proteins from normal cells (left gels) and cancer cells (right gels) treated with rabeprazole (upper) or auranofin (bottom). Lanes 4: controls, lanes 5 to 7 increasing concentrations of rabeprazole or auranofin, respectively. The polarity of the gels is indicated on the left side of each figure. (**c**) Cellular HsTIM enzyme activity and viability of normal and cancer cells treated with the drugs. Results are expressed as percentages with the values obtained in the absence of the drugs set as 100%. The results are the mean of four independent experiments. Full-length gel and blots of (**a**) and (**b**) panels are shown in Suppl. Fig. S3a,b.

HsTIM from HMECs showed the same cleavage pattern found for recombinant nondeamidated HsTIM (Fig. 2a, lane 6 *vs* lane 3), whereas HsTIM from MDA-MB-231 cells showed a cleavage pattern similar to that of recombinant HsTIM deamidated at position 16 (Fig. 2a, lane 7 *vs* lane 4). The cleavage selectivity of the hydroxylamine method was reinforced by the absence of a cleavage pattern for the recombinant twice deamidated HsTIM at positions 16 and 72 (Fig. 2a, lane 5). Considering this evidence, we demonstrated that deamidated HsTIM was present in these cancer cells rather than their normal counterparts.

Since every deamidation reaction adds one negative charge to HsTIM, native polyacrylamide gel electrophoresis (nPAGE) fulfills the requirements for analyzing the acidic *status* of both recombinant and cellular HsTIM proteins. The electrophoretic mobility patterns resulting from immunoblotting of the nPAGE gel clearly showed that cancer cells accumulated deamidated HsTIM (Fig. 2b, cancer cells, lane 4), whereas normal cells did not (Fig. 2b, normal cells lanes 4). Importantly, these two characteristics in cancer cells (absent in normal cells) strongly support the search for drugs directed to deamidated HsTIM as a cancer cell-specific target.

### Drug treatment induced the formation of inactive acidic isoforms of HsTIM in cancer cells and promoted selective cell death

Negatively charged HsTIM isoforms (acidic isoforms) appeared after the treatment of cancer cells (Fig. 2b, cancer cells, lanes 5–7) but not in normal cells (*i.e*., noncancer cells) (Fig. 2b, normal cells, lanes 5–7) with rabeprazole or auranofin. Furthermore, the naturally occurring deamidated HsTIM already present in the cancer cells tended to disappear, and other more negatively charged isoforms appeared as a result of treatment with increasing doses of both drugs (0, 500, 1000, and 1500 rabeprazole µM or 0, 25, 50, and 100 µM auranofin, respectively). Importantly, both drug treatments induced the disappearance of nondeamidated HsTIM, which potentiates the effect of the drugs on cancer cells.

Cellular HsTIM in cancer cells completely lost its enzyme activity upon treatment with 1000 µM rabeprazole, and the viability of these cells decreased by close to 80% at 1500 µM rabeprazole treatment (Fig. 2c, cancer cells, rabeprazole). Auranofin strongly affected cellular HsTIM levels in cancer cells; indeed, 250 µM auranofin completely inhibited the enzyme and decreased cell viability near to 80%. Finally, this drug at a concentration of 500 µM totally eliminated cell viability (Fig. 2c, cancer cells, auranofin). These drugs exerted effects on the enzyme activity of cellular HsTIM similar to those obtained for the recombinant enzymes.

In contrast, in normal cells, the two drugs induced no more than a 25% loss in both enzyme activity and cell viability (Fig. 2c, normal cells, rabeprazole and auranofin).

Overall, these results show that HsTIM in cancer and normal cells had opposite behavior, supporting the crucial involvement of this protein in the selective cell death exerted by these drugs.

To support the notion that the inhibition of cellular HsTIM was attributable to the drug treatments instead of the consequence of cell damage, assays with high drug concentrations and short incubation durations were performed. HsTIM activity in breast cancer cells treated with rabeprazole was almost totally abolished at 2 h of treatment, and the viability of the cells was close to 100% for 4 h until it dropped by approximately 20% in the last hour (Suppl. Fig. S1, cancer cells, rabeprazole). With auranofin, HsTIM activity was completely abolished at 4 h, whereas cell viability remained close to 100% and tended to decrease after 5 h of treatment (Suppl. Fig. S1, cancer cells, auranofin).

On the other hand, HsTIM activity in normal cells dropped by no more than 30% with rabeprazole treatment, while auranofin treatment exerted a maximum inhibitory effect of 10%. The viability of these cells was maintained close to 100% during treatment (Suppl. Fig. S1, normal cells).

These results support the notion that TIM in cancer cells is a cell-specific target of both drugs that is inactivated prior to cell death, whereas TIM in normal cells is practically unaffected. To assess the hypothesis that the drugs direct their effect to regions other than the catalytic site, the kinetics of cellular HsTIM were determined for the latter assayed conditions in untreated and treated cells. The *K*_*M*_ values for TIM in untreated and treated cancer cells were similar; however, the *V*_*max*_ values were 3.3 (with rabeprazole) and 2.6 (with auranofin) times lower than those in untreated cells (Suppl. Fig. S2 and Suppl. Table S2). Consistent with previous reports^12,21^, the *K*_*M*_ values of TIM in normal cells, either untreated or treated with rabeprazole and auranofin, were similar (Suppl. Fig. S2, and Suppl. Table S2). These results support the idea that these drugs do not compete with the active site of HsTIM and that their mechanism of inactivation is noncompetitive.

### Targeting deamidated HsTIM decreased lactate production and caused the excessive production of methylglyoxal and AGEs in breast cancer cells

**Figure 3:**
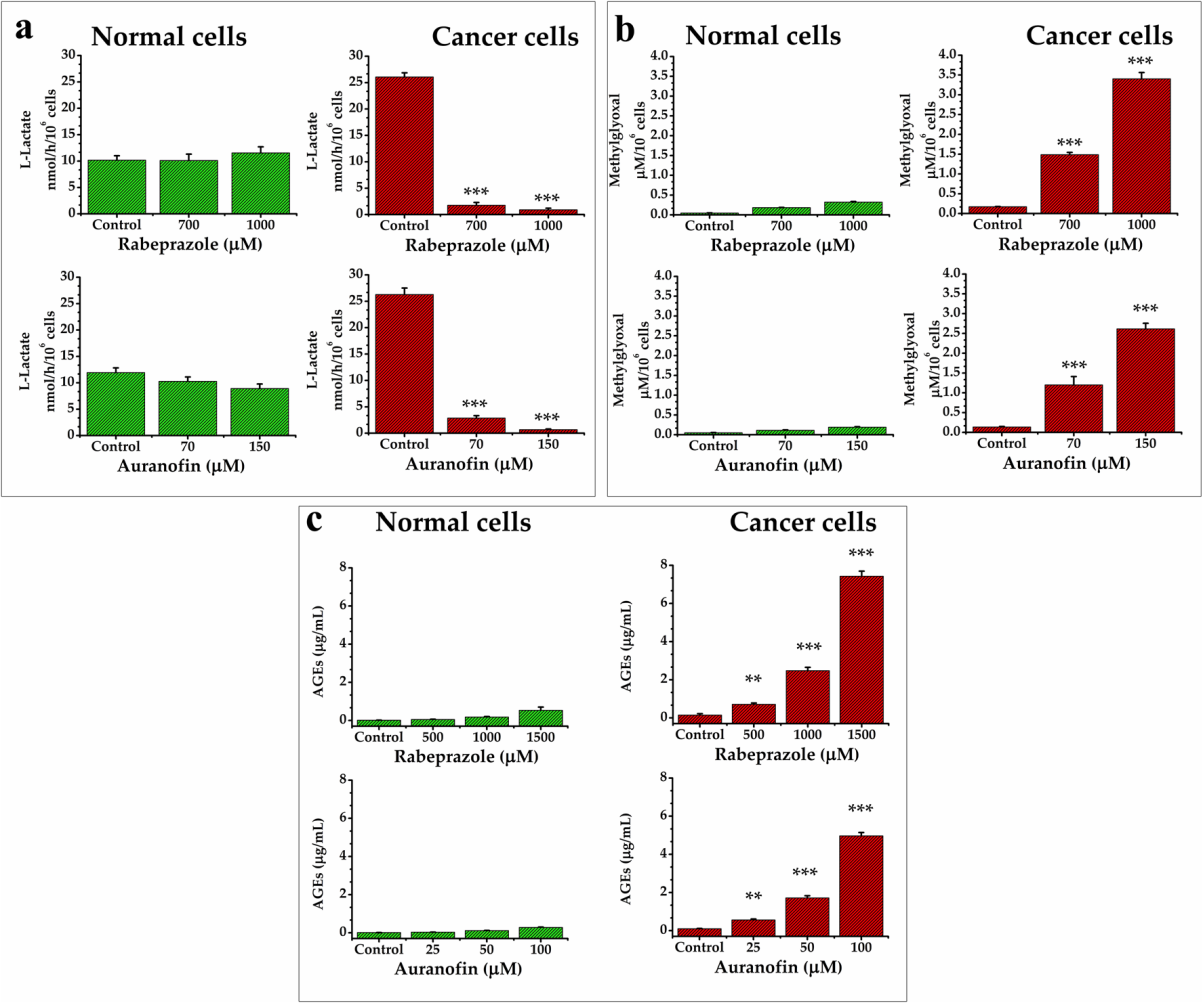
Quantification of metabolites in normal and cancer cells under drug treatments. Levels of lactate (**a**), methylglyoxal (**b**), and Advanced Glycation End products (AGEs) (**c**) in normal (green) and cancer cells (red) under rabeprazole (upper) or auranofin (bottom) treatments. Results represent the mean of at least four independent experiments. Differences among groups were assessed with one-way ANOVA and Tukey’s test with p value = 0.001 *** or with p value = 0.01**.

Targeting HsTIM affects the glycolytic pathway, which can be detected by quantifying lactate production. Breast cancer cells, which normally produce high quantities of lactate, significantly decreased their production of lactate when treated with either rabeprazole or auranofin, whereas normal cells were not affected (Fig. 3a). This supports the idea that, when HsTIM is affected by these drugs, glycolytic flux is depleted in cancer cells (Fig. 3a, cancer cells). Additionally, the malfunctioning of HsTIM causes the accumulation of DHAP, the spontaneous degradation of which leads to methylglyoxal (MGO) formation, which in turn inhibits glycolytic flux^22^.

An increase in MGO is related to HsTIM failure, as has been documented in several human disorders^23^. Concordantly, our results showed that the assayed treatments increased the production of MGO, but such production was significantly higher in cancer cells under treatment regimens than in untreated cells (control) (Fig. 3b, cancer cells). MGO is harmful to several cell types, mainly as it is an important intermediate in the formation of AGEs.

Both drug treatments significantly increased the levels of AGEs in a dose-dependent manner in cancer cells (Fig. 3c, cancer cells) but not in normal cells, in which these products were present at minimal concentrations (Fig. 3c, normal cells). These results are consistent with those observed for MGO formation upon the treatments. Hence, the behavior of deamidated HsTIM leads to the production of toxic metabolites (*i.e.,* MGO and AGEs), which in turn should be involved in the selective cell death process that we observed.

In an attempt to strengthen our hypothesis, cancer cells were subjected to the treatment with auranofin in presence of N-acetylcysteine (scavenger molecules of MGO and AGEs). Remarkably, the decrease of MGO and AGEs levels (decreasing ~ 56%) led to the enhancement of cell viability (~ 22%) with respect to those assays with auranofin treatment alone (Suppl Fig. S4). Moreover, since auranofin is recognized to produce reactive oxygen species (ROS)^24^, we assayed the contribution of the ROS levels in the treatment with auranofin on MDA-MB-231. By using quercetin as a quencher molecule of ROS, we found a moderated enhancement of cell viability (~ 8%) with respect to those assays with auranofin treatment alone. However, the levels of MGO (Suppl. Fig. S4a) and AGEs (Suppl. Fig. S4b) remained elevated (decreasing ~ 16%) in presence of this ROS quencher. Altogether, these results indicate that the mechanisms involved in this phenomenon of cellular death are mainly promoted by the elevated levels of MGO and AGEs while ROS contributed marginally.

### Breast cancer cells treated with rabeprazole and auranofin underwent apoptosis

Suppressing apoptosis is important to tumor initiation, progression or metastasis in cancer pathologies^25^. As shown above, the drugs that we used to treat cancer cells induce their overproduction of MGO and AGEs. Related to this finding, such toxic metabolites are known to induce apoptosis^26,27^. To test the influence of the assayed treatments on cell apoptosis, we analyzed the expression of proteins related to pro- and antiapoptotic events^28^ induced through intrinsic pathways (Bax and Bcl-2)^29,30^.

**Figure 4:**
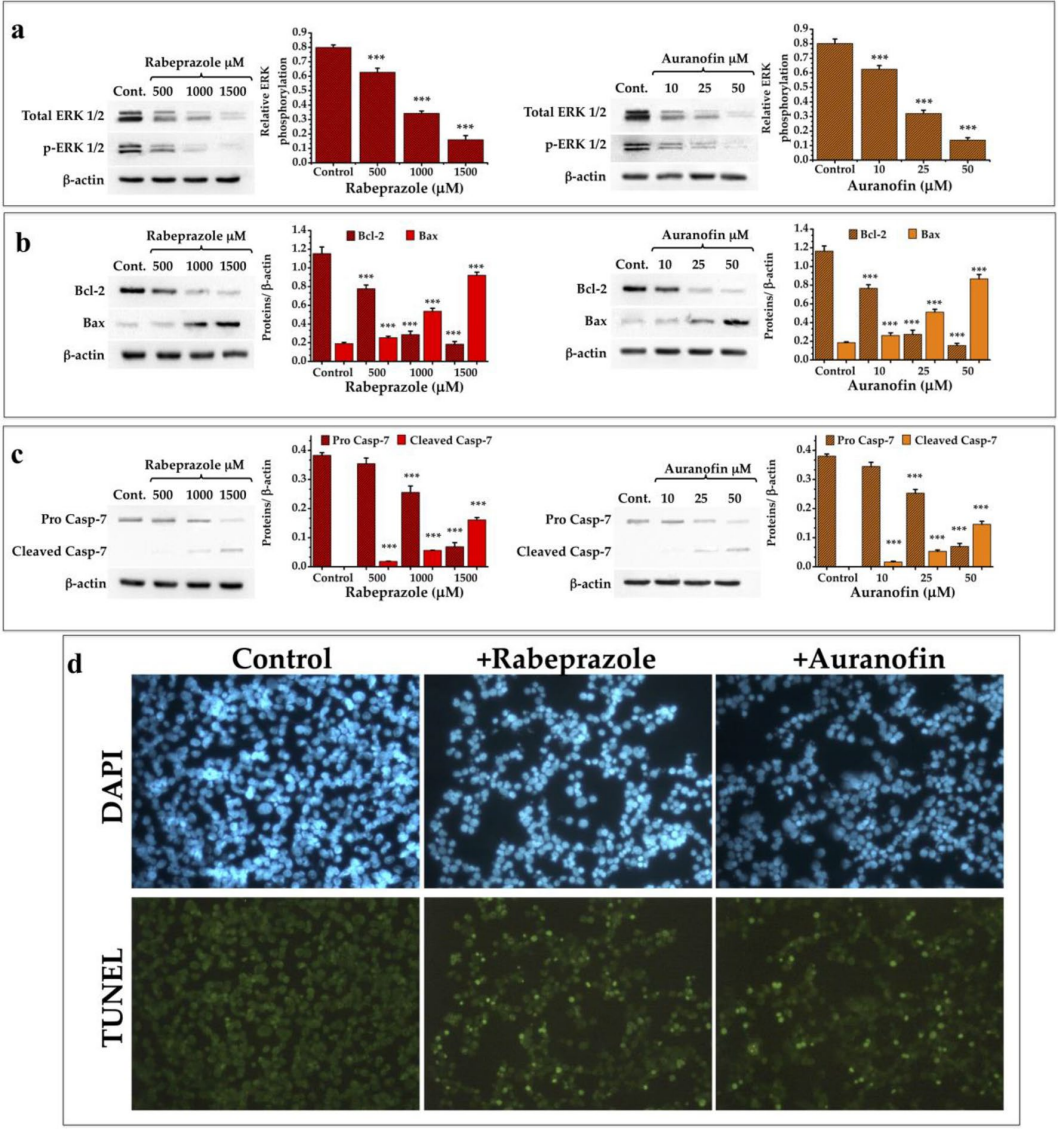
Expression of elements related to programmed cell death in cancer cells treated with thiol drugs. (**a**) Western blot analysis of the expression of ERK 1/2 and its phosphorylated form. Graphs depict the decrease of ERK 1/2 and its phosphorylated form under treatment with rabeprazole (red bars) and auranofin (orange bars). (**b**) Western blot analysis of the expression of Bcl-2 and Bax. Graphs depict the expression of these two elements under treatment with rabeprazole (red bars) and auranofin (orange bars). (**c**) Western blot analysis of the expression of pro caspase-7 and the apparition of its cleaved form. Graphs depict the relative quantities of these two forms of Casp-7 under treatment with rabeprazole (red bars) and auranofin (orange bars). β-Actin was used as a charge control. Differences among groups were assessed with one-way ANOVA and Tukey’s test with p value = 0.001 ***. (**d**) TUNEL assays of cancer cells do not treated (Control) or treated with 700 μM rabeprazole (+ Rabeprazole) or 70 μM auranofin (+ Auranofin). DAPI was used for nuclei staining. Photographs are at ×40. Full-length blots of (**a**–**c**) panels are shown in Suppl. Fig. S5.

The results showed that both drug treatments induced a decrease in the expression of total ERK 1/2 and its phosphorylated form (Fig. 4a). Additionally, the expression of Bcl-2 decreased (Fig. 4b), whereas the proapoptotic elements Bax (Fig. 4b) and cleaved procaspase-7 (Fig. 4c) were increased after both treatments in a dose-dependent manner. Transferase dUTP nick end labeling (TUNEL) assays with breast cancer cells treated with rabeprazole or auranofin showed an increase in apoptotic cells after 24 h of treatment (Fig. 4d). These results show that our strategy overcomes the antiapoptotic tendencies of cancer cells.

### Deamidated HsTIM naturally occurs in tumoral breast cancer tissues and is efficient as a druggable target

**Figure 5:**
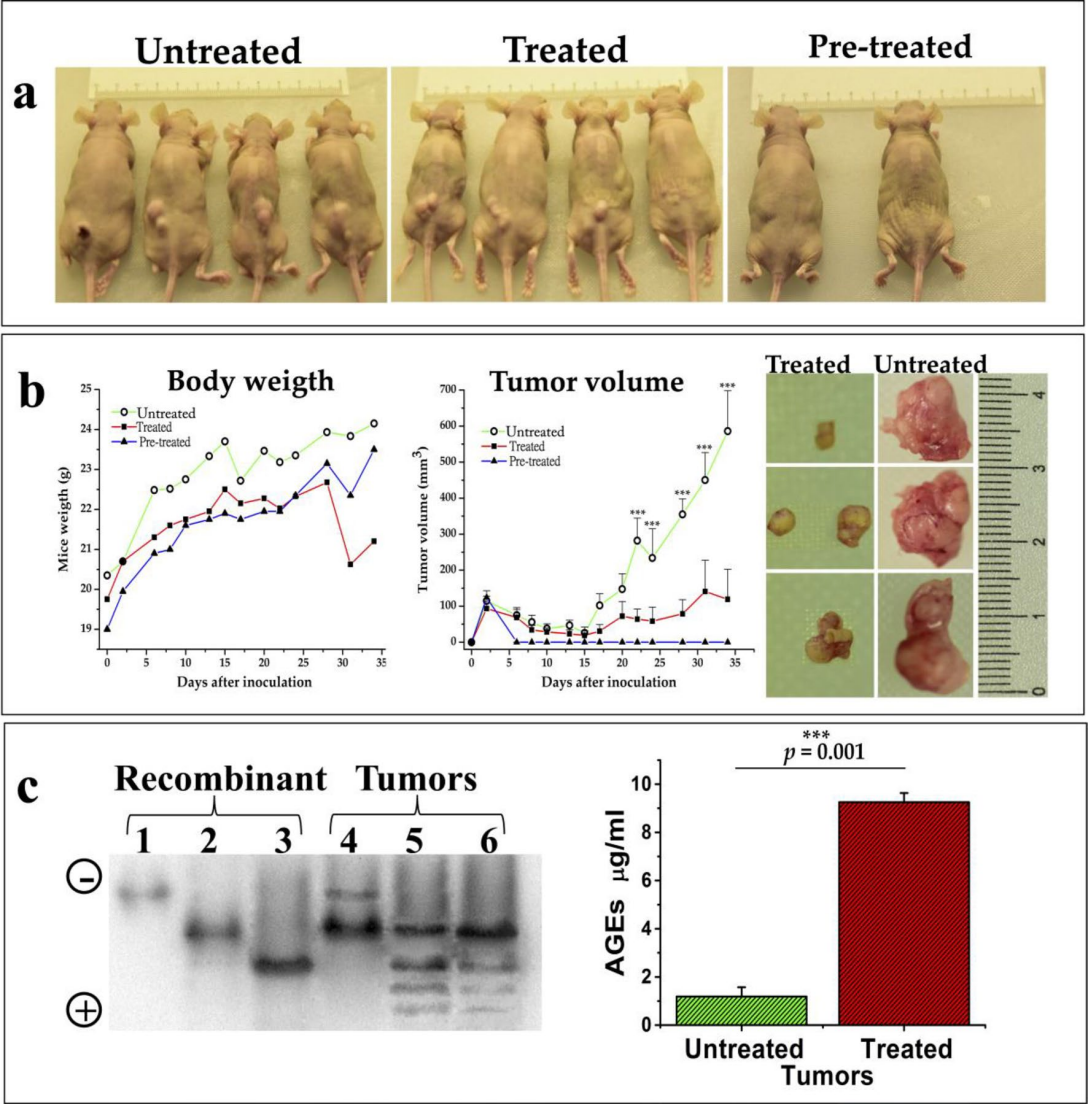
Xenograft rodent model to analyze deamidated HsTIM accumulation in tumors and the effect of rabeprazole. (**a**) Representative images of mice from the different experimental groups. Mice were subcutaneously inoculated with MDA-MB-231 cells (20 × 10^6^ cells); 7 days after cell implantation, placebo (PBS) (untreated group) or rabeprazole (50 mg/kg of weight) (treated group) were administered three times a week by intraperitoneal injection. Also, a group of mice implanted with breast cancer cells previously incubated for 24 h with 1 mM rabeprazole (pre-treated) was included. (**b**) Changes in body weight and tumor volume were noted throughout the experiment (graphs). Representative images of tumors from treated and untreated mice at the end of the experiment. (**c**) Western blot of the total proteins extracted from tumors and analyzed by nPAGE shows deamidated HsTIM and its acidic isoforms produced under the treatment with rabeprazole. Lanes 1–3, recombinant proteins of the nondeamidated (lane 1), once deamidated (lane 2), and twice deamidated HsTIM (lane 3). Lanes 4–6, the acidic isoforms of HsTIM from the xenograft tumors of untreated mice (lane 4) and two different tumors from treated mice (lanes 5 and 6). The polarity of the gel is indicated on the left side. The graph shows the significantly increased production of AGEs in tumors treated with rabeprazole (red bar) compared to untreated tumors (green bar). Differences among groups were assessed with one-way ANOVA and Tukey’s test with p-value = 0.001 ***. The full-length blot of panel “**c**” is shown in Suppl. Fig. S6.

To determine whether the properties of deamidated HsTIM observed in vitro would translate into an efficient anticarcinogenic strategy, human breast cancer cells were implanted in nude mice and treated with rabeprazole. Each mouse was subcutaneously inoculated with MDA-MB-231 cells (20 × 10^6^ cells) and 7 days after cell implantation, placebo (PBS) (untreated group) or rabeprazole (50 mg/kg of weight) (treated group) were administered three times a week by intraperitoneal injection (Fig. 5a). Pre-treated group corresponded to mice implanted with breast cancer cells previously incubated for 24 h with 1 mM rabeprazole (Fig. 5a). Mice were euthanized 34 days after inoculation of cancer cells to dissect tumors and to extract total proteins from them.

Tumorigenesis was significantly inhibited by treatment with rabeprazole three times weekly and totally inhibited when pretreated cancer cells were implanted (Fig. 5b). The most notable results from these series of experiments were (a) the finding that deamidated HsTIM naturally accumulated in the tumorigenic cells and (b) the identical electrophoretic mobility behaviors of tumorigenic HsTIM obtained in vitro and treated with rabeprazole combined with the high production of AGEs in tumors from treated mice (Fig. 5c). These findings indicate that our hypothesis can be tested in preclinical studies.

### Caspase-1 inhibition is related to the accumulation of cellular deamidated HsTIM

**Figure 6:**
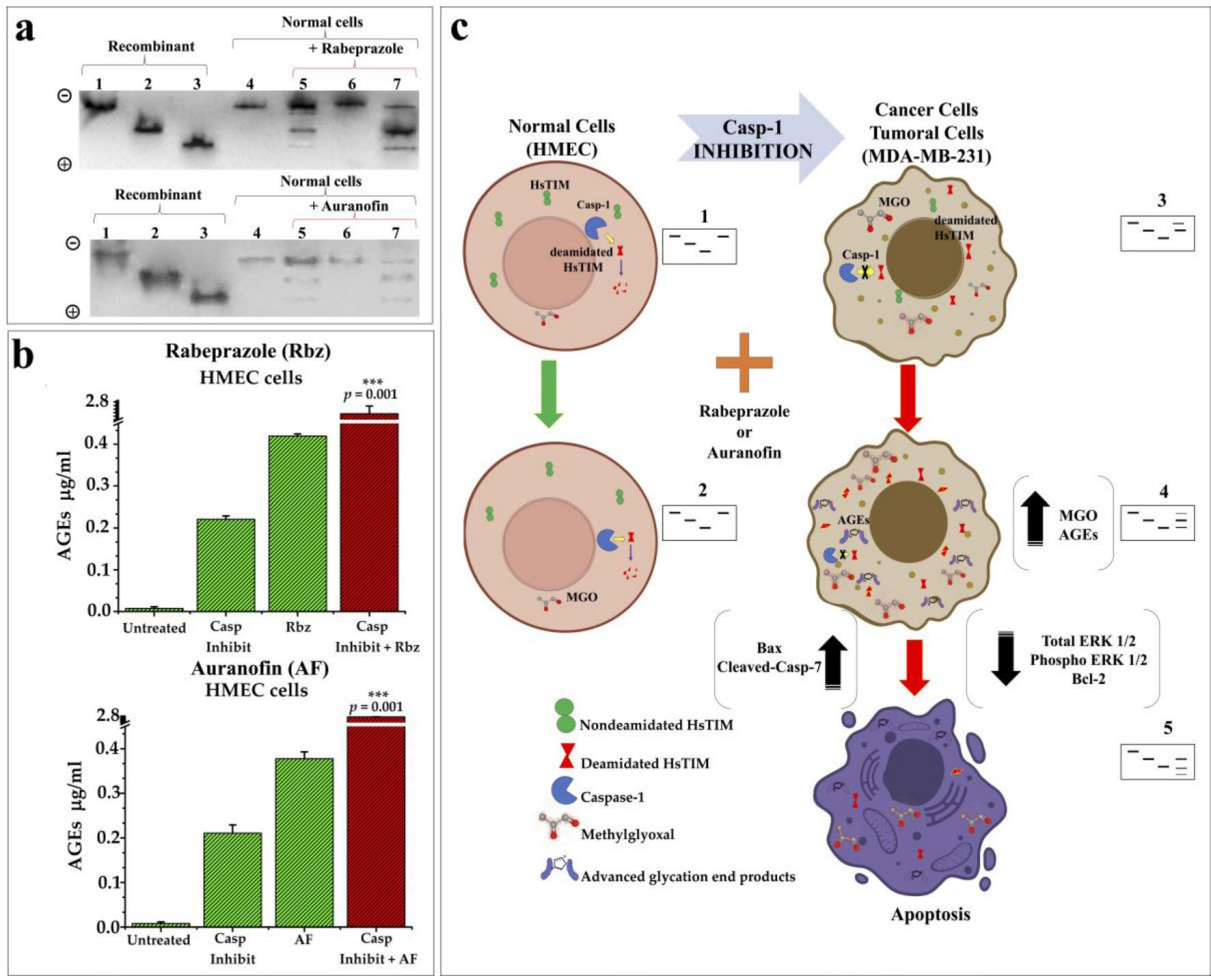
Effects of caspase-1 inhibition and thiol drugs in normal cells. (**a**) nPAGE and western blot analysis of normal cells (HMEC) with or without treatment with a caspase-1 inhibitor and thiol drugs. Lanes 1 to 3 (1 µg protein/lane): recombinant nondeamidated, once deamidated and twice deamidated HsTIM, respectively. Lanes 4 to 7 (100 µg protein/lane): proteins from the cellular extract of HMEC cells. Lanes 4: control cells without caspase-1 inhibitor and drugs treatment; lanes 5: cells incubated with the caspase-1 inhibitor; lanes 6: cells treated only with rabeprazole (top) or auranofin (bottom), lanes 7: cells incubated with the caspase-1 inhibitor and treated with rabeprazole (top) or auranofin (bottom). The polarity of the gels is indicated on the left side. (**b**) Production of AGEs in normal cells exerted by caspase-1 inhibitor and thiol drugs. Graphs show the differences on AGEs levels in normal cells by using caspase inhibitor (Casp Inhibit), rabeprazole (Rbz), or auranofin (AF) and their combined effects (Casp Inhibit + Rbz or + AF). (**c**) Schematic illustration of the possible role of deamidated HsTIM and caspase-1 in normal and cancerous phenotype. 1, 2, 3, 4, and 5 represent the corresponding nPAGE profiles of HsTIM acidic isoforms from each cell phenotype. Full-length blots of “**a**” panel are shown in Suppl. Fig. S7.

As a challenge to confirm our proposal, a biological scenario where noncancerous cells would behave as cancer cells would be overwhelming. On this line, unlike the majority of proteins, HsTIM and some other glycolytic enzymes are substrates of the protease caspase-1^31^. Certainly, according to relative caspase-1 cleavage specificity, the deamidated sequence of HsTIM (X-Asp-Gly-X) is 152 times more prone to be cleaved by Caspase-1 than its nondeamidated counterpart (X-Asn-Gly-X)^32,33^. In addition, caspase‑1 mRNA expression was found to be significantly decreased in the breast cancer tissues of patients, and treatment with a caspase-1 inhibitor markedly increased the proliferative and invasive abilities of MDA‑MB‑231 cells^34^. The keystone of our hypothesis implicates the accumulation of deamidated HsTIM in cancer cells. Therefore, we wondered whether caspase-1 plays a role on the absence of deamidated HsTIM in normal cells in contrast with its accumulation in cancer cells. Remarkably, normal cells treated with a caspase-1 inhibitor showed the de novo accumulation of acidic isoforms similar to those previously found in cancer cells (Fig. 6a, lanes 5). Additionally, caspase-1 activity was inhibited by 77%, whereas HsTIM activity was increased by 45% in the noncancer cells (Suppl. Table S3). Moreover, the acidic isoforms of HsTIM from these cells not only adopted the behavior of their counterparts in cancer cells but also had become sensitized to rabeprazole and auranofin (Fig. 6a, lanes 7). These normal cells with inhibited caspase-1 activity underwent the aforementioned changes and significantly increased their capacity to produce advanced glycation end products (AGEs) when they were treated with rabeprazole or auranofin (Fig. 6b). Based on these data, our results show how normal cells can be guided toward “cancer-like” behavior regarding glycolysis, showing characteristic HsTIM nPAGE profiles, and leading to selective cell apoptosis (Fig. 6c). Altogether, the results support the mechanisms involved in the natural accumulation of deamidated HsTIM in cells, leading to the generation of a selective target.

## Discussion

Here we report the selective effect of rabeprazole and auranofin on HsTIM in vitro, in situ, and in vivo. The effects exerted by these drugs are guided by the derivatization of Cys residues (Fig. 1c, Suppl. Table S1). Cancer targets are usually selected based on their enhanced expression or even their overactivity^35,36^; on this basis, human TIM fulfills one requirement for use as a drug target as it is upregulated in cancer cells^4,37^. Nevertheless, this protein is present in all cells regardless of whether they are cancerous, which importantly diminishes the cellular selectivity of the chosen drug. To address this issue, we propose a new approach to enhance drug selectivity based on the distinctive characteristics of deamidated HsTIM. First, the presence of deamidated HsTIM in cancer cells but not in their normal counterparts was demonstrated with a selective cleavage method and nPAGE (Fig. 2a,b, respectively). Second, deamidated HsTIM was shown to accumulate in cancer cells, as demonstrated by the identification of the corresponding acidic isoform (Fig. 2b). Third, we showed the role of deamidated HsTIM in cancer cell-specific cell death induced by rabeprazole and auranofin (Fig. 2c). The selective inhibition of deamidated HsTIM, de novo generation of acidic isoforms, death of the cancer cells, and inhibition of tumor growth in vivo support this.

HsTIM has been shown to be a potential target in cancer mainly by the use of inhibitors directed to its active site or factors that diminish its expression^38^. Nonetheless, the main problem with these proposals is that the molecular elements involved in the likely therapeutic strategies are present at both, pathologic and normal stages. Consequently, an important disadvantage of the latter strategy is that it largely reduces the safety of such methods. Herein, we have demonstrated that deamidated HsTIM is a distinctive and conspicuous target in breast cancer cells but not in their noncancerous counterparts. Hence, our proposal would be helpful to contribute to the drug design pipeline in the pharmaceutical industry and to increase the success rate in clinical trials.

MDA-MB-231 cells are triple-negative breast cancer cells characterized by the minimal expression of estrogen and progesterone receptors and absence of human epidermal growth factor receptor-2 (HER2). Because these cancer cells lack HER2, hormone therapy and drugs targeting HER2 are not helpful, leaving chemotherapy as the main systemic treatment option for triple-negative breast cancer. The main therapies used for this cancer combine the drugs adriamycin and cyclophosphamide; adriamycin, cyclophosphamide, and paclitaxel; and docetaxel and cyclophosphamide^39^. Since these drugs are directed to targets (mainly nucleic acids and the replication machinery) present in both cancer and normal cells, the side effects of such treatments are considerable and usually severe. Additionally, their economic burden has a great impact on patients suffering from this disease^40^. In contrast, our strategy would represent a safer and less expensive therapy for this kind of cancer.

It is important to note that the two different drugs used yielded the same output, especially that caused by the effects on deamidated HsTIM. Accordingly, both treatments exert selective cellular death, largely attributable to the presence of deamidated HsTIM and its effect in these cancer cells. Consequently, glycolytic flux diminishes, and the concentrations of MGO and AGEs concomitantly increase. Although energy metabolism was affected, the main cause of selective cell death was the toxic effect of MGO overproduction, as shown in other diseases^41,42^. Albeit auranofin is recognized to produce (ROS)^24^, and high concentrations of ROS can cause cancer cell apoptosis^43^, our results using a quencher of ROS strongly support that such reactive species is not the main cause of cell death observed in our experimental model. In fact, by using an MGO scavenger we were able to demonstrate that the observed cell death is tightly related to the production of MGO and AGEs, as has been pointed out in the rest of the experiments herein.

Mounting evidence indicates that MGO and AGEs can induce the apoptosis process^44,45^. This was corroborated in our study as the expression of ERK1/2 and its phosphorylation were decreased, accompanied by a decrease in Bcl-2, whereas the expression of Bax was increased and the executing apoptosis factor caspase-7 was activated in a dose-dependent manner. TUNEL assays corroborated that the observed type of cell death was apoptosis.

Since the response of cancer cells in tumors is affected by factors such as their heterogeneity, components of the microenvironment, and anatomical structures for proper growth^46^, we translated our study to a xenograft model. Importantly, in this preclinical scenario, the tumor size was significantly smaller in treated mice, and tumors were absent in pretreated mice. Most important in the context of validating the protein concerned was corroborating the accumulation of acidic isoforms equivalent to deamidated HsTIM in all tumors. Importantly, as a result of drug treatment, acidic isoforms were generated de novo, HsTIM enzyme activity was inhibited (Suppl. Table S4), and the AGEs concentration increased (Fig. 5c). Collectively, these results in tumors faithfully mimicked the behavior of HsTIM demonstrated in cell lines. Other proton pump inhibitors (PPIs) have been reported to act as tumor suppressors^6,47^, with proteins upregulated in cancer stages acting as possible targets; nonetheless, their greatest weakness is that the proposed targets are found in both cancer and normal cells.

The role of deamidated HsTIM as a triple-negative breast cancer-specific target was supported by the change in phenotype of normal cells (HMECs) to a cancer phenotype, based on the described HsTIM behavior. This was achieved by inhibiting cellular caspase-1 activity in HMECs (Suppl. Table S3). Most importantly, these cells were sensitized to the drug treatments (Fig. 6). Indeed, we previously demonstrated in a cell model that the presence of deamidated HsTIM alone caused sensitization to another PPI, impairing cell growth^10^.

The strategy stated here highlights the posttranslational modifications favored in cancer cells, which naturally generate cancer-specific targets. Based on the knowledge acquired herein, pathologic cells should contain a collection of proteins with special skills similar to those shown by HsTIM, that is, structural signatures that usually promote changes in proteins that drive their dead-end elimination (i.e., protein turnover) in normal cells but translate into new functions that lead to adaptive advantages in pathological cells. MGO is a hormetic metabolite related to tumor growth and metastasis^48,49^. In fact, in this effect, we found a hidden opportunity to push the triple-negative cancer cells to overproduce MGO and commit them to suicide (apoptosis); achieving with this, a novelty and safer strategy on the therapeutics for a such highly aggressive cancer. In contrast, cell death would not be as specific for malignant cells if toxic metabolites were administered exogenously. Our approach could also be adapted to different proteins that meet the characteristics described herein and to other types of cancer or even to other pathologies.

## Methods

All experimental protocols were approved by the Investigation, Ethics and Biosecurity Committees of the National Institute of Pediatrics (Protocol numbers: 2019/072 and 2020/016); and the use of mice was approved by the Experimental Animal Ethics Committee of National Autonomous University of Mexico, Faculty of Chemistry, Mexico (protocol number: UNIPREC-19-020).

All Experiments were performed according to the relevant guidelines and regulations. The use of animals was conducted according to federal and local ethical laws and complied with the ARRIVE guidelines.

### Reagent and general materials

Luria–Bertani (LB) medium and isopropyl-β-d-thiogalactopyranoside (IPTG) were purchased from VWR Life Science Products (Radnor, Pennsylvania, USA). Glycerol-3-phosphate dehydrogenase (α-GDH), l-lactate dehydrogenase from rabbit muscle and reduced nicotinamide adenine dinucleotide (NADH) were obtained from Roche (Penzberg, Upper Bavaria, Germany). Immobilized metal affinity chromatography (IMAC) resin was obtained from Bio-Rad (Hercules, California, USA). Sephadex G-25 Fine Resin was obtained from Amersham Biosciences (Amersham, UK). Amicon Ultra 30 kDa filters were purchased from Merck-Millipore Corporation (Billerica, Massachusetts, USA). Fetal bovine serum (FBS), penicillin, streptomycin and trypsin EDTA solutions were purchased from Invitrogen (Carlsbad, USA). The other reagents mentioned were acquired from Sigma-Aldrich (St. Louis, MO, USA).

### Molecular docking studies of deamidated and nondeamidated HsTIM

To carry out the docking studies, the crystallographic structures of HsTIM WT (nondeamidated) and HsTIM N16D (deamidated) that had been deposited in the Protein Data Bank (PDB) were downloaded. Atomic coordinates of the nondeamidated and deamidated proteins (PDB IDs: 2JK2 and 4UNK, respectively) were prepared by removing all water molecules and heteroatoms with PyMOL version 2.0.7 (Schrödinger Inc, NY, USA). The structures were energy minimized with Chimera software^50^, and the new coordinates were used for docking calculations. Structures of the drugs rabeprazole and auranofin were obtained from the PubChem Compound Database (https://pubchem.ncbi.nlm.nih.gov) and energy minimized with Avogadro version 1.2. Structures of the nondeamidated and deamidated HsTIMs were prepared by adding hydrogen atoms and Kollman charges (6.00 and 3.999, respectively) with AutoDock Tools (ADT) version 1.5.6^51^. The molecular docking program AutoDock Vina version 1.1.2^52^ was used with the default settings, and the output files were saved in pdbqt format. Protein receptors and ligands were converted into pdbqt format. The ligand-binding site was defined as the interface of the dimer. After docking, close interactions for binding of the target with the ligands were analyzed and visualized using ADT and PyMOL version 2.0.7.

### Expression and purification of recombinant enzymes

The genes encoding wild-type and mutant (WT, N16D and N16D/N72D) HsTIM were cloned into the vector pET3a-HisTEV as previously reported^12^. The plasmid provides six histidine residues (His6) at the N-terminus of the protein and a tobacco etch virus protease (TEVp) recognition sequence that facilitates protein purification. The plasmids containing inserts (pET3a-HisTEV-wt-HsTIM, pET3a-HisTEV-N16D-HsTIM and pET3a-HisTEV-N16D/N72D-HsTIM) were used to transform the *Escherichia coli* BL21-CodonPlus-RIL strain. The overexpression and purification of recombinants WT (nondeamidated HsTIM), N16D HsTIM (deamidated HsTIM) and N16D/N72D HsTIM (twice deamidated HsTIM) were carried out as previously described^12^. Purified proteins were ultrafiltered with Centricon filters (cutoff of 30 kDa for WT and 10 kDa for N16D and N16D/N72D) until the volume reached 0.5 mL, which was repeated 3 times after the addition of 5 mL of buffer containing 100 mM triethanolamine and 10 mM EDTA (pH 7.4) (TE buffer). Finally, the concentrated proteins were precipitated with ammonium sulfate at 75% saturation and maintained at 4 °C. To remove the His_6_-TEV tag, the protein suspension was centrifuged at 12,000 rpm for 20 min at 4 °C, and the pellet was resuspended in 50 mM Tris (pH 8.0), 0.5 mM EDTA and incubated at room temperature for 16 h in the presence of the protease TEVp at 1:50 (w/w) (protease/HsTIM) and 1 mM dithiothreitol (DTT). Thereafter, the incubated sample was loaded into a column containing IMAC resin previously equilibrated with 100 mM triethanolamine (pH 7.4). Enzymes without the His_6_-TEV tag were recovered, ultrafiltered, precipitated with ammonium sulfate and stored at 4 °C until use. To remove the precipitating agent, the protein was centrifuged as mentioned and suspended in TE buffer. The protein concentration was calculated spectrophotometrically at 280 nm with an extinction coefficient of ε = 33,460 M^−1^ cm^−1^^53^. The purity and integrity of the proteins were verified by sodium dodecyl sulfate polyacrylamide gel electrophoresis (16% SDS-PAGE), and proteins were stained with colloidal Coomassie brilliant blue.

Prior to any assays, the recombinant enzymes were equilibrated in TE buffer and incubated in the presence of 5 mM DTT for 30 min at 4 °C. To remove the reducing agent, the protein was spin filtered in a 1-mL column loaded with Sephadex G-25 Fine Resin previously equilibrated with TE buffer, and the protein concentration was estimated by the absorbance at 280 nm.

### Inactivation assays with nondeamidated (WT) and deamidated (N16D) HsTIM treated with rabeprazole and auranofin

Recently, we demonstrated that the PPI omeprazole selectively inactivates deamidated HsTIM^10^. Furthermore, in a previous work, we showed that among commonly used PPIs (benzimidazole derivatives), rabeprazole was the most efficient PPI in inactivating the TIM of *Giardia lamblia*^54^. Therefore, we chose rabeprazole based on the latter finding; additionally, we tested auranofin to compare the effects of rabeprazole with those of a non-PPI drug. A 100 mM stock solution of rabeprazole was prepared; to acid activate it, the solution was solubilized in 20% dimethyl sulfoxide and 5% 0.1 N HCl, incubated for 30 min at room temperature in the dark, and diluted with TE buffer to obtain a 5 mM solution. A 100 mM stock of auranofin was prepared by dissolving auranofin in 100% ethanol, and serial dilutions were made with TE buffer. For the inactivation assays, the recombinant enzymes were incubated at 0.5 mg/mL for 2 h at 37 °C in the presence of 0, 10, 25, 50, 100 and 250 µM rabeprazole or auranofin. After incubation, the samples were diluted, and 5 ng/mL and 50 ng/mL samples of WT (non deamidated) and N16D (once deamidated), respectively, were taken to measure their enzymatic activity. Enzyme activity was spectrophotometrically measured (Spectrophotometer Cary 50, Agilent Technologies, CA, USA) by following DHAP synthesis with a coupled system that followed the oxidation of NADH at 340 nm^55^. The results represent the mean of four independent experiments and are expressed as the percent activity *versus* drug concentration, with the activity of the enzyme without the drug set at 100%.

### Quantification of free thiols in recombinant enzymes

Because the principal mechanism of action of the drugs employed here (rabeprazole and auranofin) is thought to be the derivatization of Cys residues, the number of derivatized Cys residues in the recombinant enzymes was determined by using Ellman’s reagent (5,5′-dithiobis-(2-nitrobenzoic) acid, DTNB)^56^. To carry out the experiment described above, 0.5 mg/mL protein was incubated without or with 250 and 50 µM rabeprazole and auranofin, respectively, for 2 h at 37 °C. After the incubation period, the proteins were extensively washed by ultrafiltration with Centricon filters to eliminate excess drug, and the protein concentration was estimated by determining the absorbance at 280 nm. Next, an aliquot was taken and used to evaluate the residual activity of assayed proteins. The free thiol (Cys) content of the samples was spectrophotometrically quantified as follows: the basal absorbance of 1 mM DTNB and 5% SDS dissolved in TE was measured at 412 nm (ε _412 nm_ = 14.1 mM^−1^·cm^−1^), and the increase in absorbance following the addition of 200 µg of protein was monitored. The number of derivatized Cys residues was indirectly calculated by subtracting the number of free Cys residues in the derivatized enzyme (treated with rabeprazole or auranofin) from the number of free Cys residues in the enzyme in the absence of the drugs. The results represent the mean of at least four independent experiments.

### Determination of fluorescence emission spectra of the recombinant enzymes

The intrinsic and extrinsic fluorescence emission spectra of the proteins were measured using an LS55 spectrofluorometer (Perkin Elmer, Waltham MA, USA). WT or N16D HsTIM (0.5 mg/mL) was incubated for 2 h at 37 °C with or without 250 and 50 µM rabeprazole and auranofin, respectively. Next, the proteins were extensively washed by ultrafiltration with Centricon filters to eliminate excess drug, and their concentration were recalculated by measuring their absorbance at 280 nm. To determine the intrinsic fluorescence, the enzymes (0.5 mg/mL) were excited at 295 nm, and the fluorescence emission spectra were recorded from 300 to 500 nm. To determine the extrinsic fluorescence, a stock solution of 15 mM ANS dissolved in methanol was prepared. Then, 100 µM ANS was added to the samples (maintained with slow agitation), which were excited at 385 nm, and the fluorescence emission spectra were monitored from 400 to 600 nm. For each sample reading, the background fluorescence was subtracted (buffer with drug, buffer with ANS, or buffer with ANS and rabeprazole or auranofin). Each spectrum is the average of three scans. The results represent the mean of four independent experiments and are expressed as the percent fluorescence intensity *versus* wavelength.

### Cell culture and identification of cellular deamidated HsTIM by the selective cleavage of Asn-Gly

For general cell culture procedures, the MDA-MB-231 (breast cancer cells) cell line was obtained from American Type Culture Collection (ATCC; Rockville, MD, USA) and maintained in DMEM supplemented with 10% FBS and antibiotics (100 U/mL penicillin and 100 mg/mL streptomycin). Acting as noncancerous (control) cells, HMECs were obtained from ATCC and cultured in mammary epithelial cell basal medium (ATCC #PCS-600-030) supplemented with components of the mammary epithelial growth kit (ATCC #PCS-600-040). For all experiments, both cell lines were used from passages 2–5 and cultured at 37 °C in a humidified 5% CO_2_ atmosphere, detached with 0.05% trypsin in 0.53 mM EDTA, incubated for 5 min at 37 °C, centrifuged at 2000 rpm for 5 min and washed three times with phosphate-buffered saline (PBS).

Since the amino acid sequence of HsTIM contains two Asn-Gly pairs, both cell lines were properly maintained and used to determine the deamidation of cellular HsTIM by selective cleavage with hydroxylamine^18^. A validated method for peptide mapping and sequence analysis of Asn-Gly pairs^19^, this method is based on placing the proteins under alkaline conditions (*i*.*e*., pH 9.0) to deprotonate the amide group of the Asn residue, which in turn exert a nucleophilic attack on the adjacent Gly residue located in the C-terminus. This results in the formation of succinimide, which is selectively cleaved in the presence of an excess of hydroxylamine. Notably, deamidated proteins contain Asp (or isoAsp) instead of Asn; thus, under the conditions described for this method, succinimide is not formed, preventing cleavage by hydroxylamine^20^.

HMECs (1 × 10^7^) and MDA-MB-231 cells were incubated for 24 h under the conditions mentioned above. After washing, the cells were lysed in ice-cold RIPA lysis buffer containing protease inhibitors (sc-24948, Santa Cruz Biotechnology, Santa Cruz, CA, USA) and centrifuged at 12,000 rpm for 20 min at 4 °C. The supernatant was collected, and the protein content was quantified by Bradford assay. The protein extracts from each culture were immunoprecipitated with anti-human TIM (H-11) (Santa Cruz Biotechnology) for 1 h at 4 °C. After this, protein A/G agarose (Santa Cruz Biotechnology, sc-2003) was added to the mixtures, which were adequately resuspended and incubated overnight at 4 °C. The mixtures were centrifuged at 2000 rpm for 3 min and washed with PBS 7 times. The immunoprecipitation products were washed with 0.2 M glycine–HCl to separate the protein A/G plus protein-antibody-protein complex. To obtain the isolated cellular HsTIM, samples were centrifuged at 2000 rpm for 5 min, immediately after which the supernatant was taken, and the pH was neutralized with Tris buffer (pH 8.0).

Hydroxylamine cleavage of recombinant and cellular HsTIM was performed as follows. WT (nondeamidated), N16D (once deamidated) and N16D/N72D (twice deamidated) recombinant HsTIM enzymes and cellular HsTIM (1 mg/mL) were separately incubated for 8 h at 45 °C with 2 M hydroxylamine-HCl and 1 M sodium carbonate (Na_2_CO_3_), pH 9.0. Immediately after incubation, the samples were loaded on a 16% SDS-PAGE gel. To analyze the patterns of HsTIM hydrolysis, gels were stained with colloidal Coomassie brilliant blue. The assays were carried out in triplicate to guarantee reproducibility.

### Native gel electrophoresis and western blot analysis of recombinant enzymes, protein cellular extracts, and tumors

The HsTIM isoforms from cellular extracts were identified with nPAGE combined with western blot analysis using the monoclonal antibody anti-TIM (H11). HMECs or MDA-MB-231 cells (1 × 10^7^) were exposed to 0, 500, 1000, and 1500 µM rabeprazole and 0, 25, 50 and 100 µM auranofin for 24 h under the above mentioned conditions. Cells were lysed in ice-cold RIPA lysis buffer containing protease inhibitors. Protein quantification was performed by the Bradford method. nPAGE gels were loaded with 1 μg/lane recombinant nondeamidated, N16D (once deamidated), and N16D/N72D (twice deamidated) HsTIM proteins and 100 μg/lane cellular protein extracts. nPAGE gels were prepared with 7% polyacrylamide and Tris–glycine buffer at pH 8.5^57^. Samples were mixed with native buffer and run at 7 mA and 4 °C for 3 h. Proteins were transferred to polyvinylidene difluoride (PVDF) membranes (0.8 mA/cm^2^, 2 h) in 25 mM Tris buffer containing 192 mM glycine and 20% methanol. The membrane was blocked for 1 h with Tris-buffered saline with 0.1% Tween-20 (TBS-T) supplemented with 5% bovine serum albumin (BSA), washed one time with TBS-T and incubated overnight at 4 °C with anti-TIM (H-11) diluted 1:3000 in TBS-T containing 1% BSA and washed three times with the same buffer. An anti-mouse IgG secondary antibody (diluted 1:5000) conjugated with horseradish peroxidase (HRP) was used to reveal the immunoblot bands by chemiluminescence (Clarity Western ECL substrate, Bio-Rad) following the supplier’s instructions. Blot image acquisition was performed using a ChemiDoc XRS + system (Bio-Rad Laboratories, Inc., USA).

nPAGE and immunoblotting in tumors were performed as follows. Tumors from nude mice were obtained by dissection and immediately frozen with liquid nitrogen. Afterwards, they were macerated with a mortar until pulverized, immediately after which they were resuspended in PBS and treated as described above. All the assays were carried out in triplicate.

### Cell proliferation and enzymatic assays to assess cultured cells under drug treatment

HMECs and MDA-MB-231 cells were exposed to drugs, after which cell proliferation and the inactivation of cellular HsTIM were determined as follows. A total of 1 × 10^5^ cells/well in a final volume of 250 µL in six-well plates were exposed to 0, 100, 250, 500, 1000 and 1500 µM rabeprazole and 0, 10, 25, 50, 100, 250 and 500 µM auranofin for 24 h under the culture conditions mentioned above. The cells were detached, washed, and quantified with a hemocytometer. Cell proliferation was measured with 3-(4,5-dimethylthiazol-2-yl)-2,5-diphenyltetrazolium bromide (MTT). HMECs or MDA-MB-231 cells (1 × 10^3^) were mixed in 100 μL of PBS per well in a 96-well plate, and 10 μL of MTT was added and incubated for 4 h in the dark. The formed formazan crystals were dissolved in DMSO, and the absorbance at 570 nm was measured in an Epoch microplate spectrophotometer (BioTek, Vermont, USA)^58^. The results are shown as the mean of four independent experiments and presented as the percent viability *vs* drug treatment; the viability measured with cellular extracts without treatment was set at 100%. To measure cellular HsTIM enzyme activity, the coupled assay system described above was used. HMECs or MDA-MB-231 cells exposed to drugs were detached, washed and resuspended in TE buffer. Cell suspensions were lysed with 5 freeze/thaw cycles (liquid nitrogen and 40 °C). Then, the protein concentration was determined by Bradford assay. For enzymatic assays, 40-µg protein samples from the cellular extracts were added to 500 µL of enzymatic reaction mixture and spectrophotometrically assessed at 340 nm. The results are presented as the percent activity *vs* drug treatment, and TIM enzyme activity in cellular extracts without treatment was set at 100%.

### l-Lactate measurements

Extracellular l-lactate levels were determined by measuring the reduction of NAD^+^ to NADH by l-lactate dehydrogenase according to Bergmeyer et al.^59^. HMECs and MDA-MB-231 cells (5 × 10^6^) were treated with 0, 700 and 1000 µM rabeprazole and 0, 70 and 150 µM auranofin for 24 h under the abovementioned culture conditions. Then, the cells were extensively washed with Krebs–Ringer buffer (125 mM NaCl, 5 mM KCl, 25 mM HEPES 1.4 mM CaCl_2_, 1 mM KH_2_PO_4_, 1 mM MgCl_2_, pH 7.4) and resuspended in 250 μL/1 × 10^6^ cells. The samples were incubated for 10 min at 37 °C under agitation. Then, a 5 mM glucose solution was added and incubated for 0 and 25 min at 37 °C under agitation; at the end of the incubation period, the cells were centrifuged, and the supernatants were carefully taken and stored at − 70 °C for the further determination of l-lactate levels. Standard values were determined from a 20 mM l-lactate stock solution in distilled water (standard curve: 0 to 500 μM). To generate the standard curve, aliquots of lactate were added to a cuvette that contained 0.4 M hydrazine, 0.5 M glycine (pH 9.5), 1 mM NAD^+^, and 20 U/mL l-lactate dehydrogenase at 25 °C. The reduction of NAD^+^ was spectrophotometrically (Cary 50 UV/Vis) recorded at 340 nm. To calculate the extracellular lactate level in the samples, 50 μL of supernatant was added to the cuvettes, and NAD^+^ reduction was recorded. Finally, the concentration of lactate was calculated using the extinction coefficient for NADH (ε = 6200 M^−1^ cm^−1^) and a standard curve. The results are shown as the mean of four independent experiments and expressed as nmol of l-lactate/h/1 × 10^6^ cells.

### Essays with auranofin in presence of scavenger molecules of reactive oxygen species (ROS), MGO and AGEs

Both, 100 μM quercetin plus 100 μM auranofin were mixed directly in DMEM culture medium (above described) and added to MDA-MB-231 (1 × 10^6^ cells). Cells cultures were incubated for 24 h in the conditions above mentioned. After incubation time, cells were extensively washed and resuspended in PBS, and cell proliferation was measured with MTT as above described.

Both, 100 μM N-acetylcysteine (NAC) plus 100 μM auranofin were mixed directly in DMEM culture medium (above described) and added to MDA-MB-231 (1 × 10^6^ cells). Cells cultures were incubated for 24 h in the conditions above mentioned. After incubation time, cells were extensively washed and resuspended in PBS, and cell proliferation was measured with MTT as above described. The assays were carried out in triplicate to guarantee reproducibility. MGO and AGEs were quantified as follows.

### MGO quantification

Intracellular free MGO was spectrophotometrically measured by using 2,4-dinitrophenylhydrazine (DNPH) according to the method of Gilbert and Brandt^60^ with modifications^61^. Briefly, 5 × 10^6^ HMECs and MDA-MB-231 cells were treated with 0, 700, and 1000 μM rabeprazole and 0, 70, and 150 μM auranofin for 24 h under the abovementioned general culture conditions. For the essays with quercetin or N-acetylcysteine, auranofin was added to a final concentration of 150 μM. Cells were resuspended in PBS and lysed with five freeze/thaw cycles, after which 0.45 M perchloric acid was added to each sample, chilled on ice for 10 min, and centrifuged at 12,000 rpm and 4 °C for 10 min. The supernatant was collected and stored at − 70 °C for further measurement. Before determining the MGO concentration in the samples, standard MGO values were calculated. Stock solutions of 20 mM DNPH in HCl-ethanol (12:88) and 0.1 mM MGO in distilled water were prepared. Increasing concentrations of MGO (0 to 10 μM) were incubated with 0.2 mM DNPH at 42 °C for 45 min; then, the samples were cooled for 5 min at room temperature, and the absorbance of MGO-bis-2,4-dinitrophenylhydrazone was recorded at 432 nm on a microplate spectrophotometer (Epoch, BioTek, Winooski, VT, USA). Finally, the cell supernatants were taken and used to quantify MGO levels with DNPH. The MGO concentrations from the cells and standard curve were estimated using the extinction coefficient ε = 33,600 M^−1^ cm^−1^ for MGO-bis-2,4-dinitrophenyl-hydrazone and the standard curve. The assays were carried out in triplicate to guarantee reproducibility. The results are expressed as [μM] MGO/1 × 10^6^ cells.

### AGEs quantification

AGEs were determined by using an AGE ELISA kit with the manufacturer’s instructions (MyBioSource, San Diego, CA, USA). HMECs and MDA-MB-231 cells (5 × 10^6^) were treated with 0, 700, and 1000 μM rabeprazole and 0, 70, and 150 μM auranofin for 24 h under the abovementioned culture conditions. After washing, cells were lysed with RIPA buffer containing protease inhibitors, and the protein extract was separated. The protein concentration was determined with the Bradford method.

The protein concentration in the samples was adjusted to 1 mg/mL, after which the samples were diluted 1:100 and loaded on ELISA plates to determine the AGE concentration. Then, avidin-peroxidase conjugates were added to ELISA wells, and 3,3′,5,5′-tetramethylbenzidine (TMB) was used as the substrate for coloring after the reactant was thoroughly washed out with PBS. Additionally, a standard curve was made with the AGE standard included in the kit. Standard concentrations were 0, 3.12, 6.25, 12.5, 25, 50, 100 and 200 ng/mL. The absorbance at 450 nm was measured within the first 10 min in an Epoch microplate spectrophotometer (BioTek, Vermont, USA). The results are the mean of four independent experiments and are expressed as μg of AGEs/mL.

### Determining the factors related to induced cell death

To assess whether the induced cell death in breast cancer cells was due to apoptosis, assays were conducted as follows. MDA-MB-231 cells (1 × 10^7^) were treated with 0, 500, 1000 and 1500 μM rabeprazole and 0, 10, 25 and 50 μM auranofin for 24 h. Then, the cells were resuspended in PBS and lysed in ice-cold RIPA buffer with protease inhibitors, and the lysates were stored at − 70 °C until use. Protein samples were loaded into 16% SDS-PAGE gels and transferred to PVDF membranes (0.8 mA/cm^2^, 2 h) in 25 mM Tris buffer, 192 mM glycine and 20% methanol. After this, nonspecific binding sites were blocked with TBS-T and 5% BSA, and the membranes were washed one time with TBS-T and incubated overnight at 4 °C with primary antibodies directed against ERK 1/2 (C-9), p-ERK1/2 (12D4), Caspase-7 (10-1-62), Bcl-2 (C-2), Bax (B-9) and β-Actin (C-2) (Santa Cruz Biotechnology, Santa Cruz, CA, USA) at a dilution of 1:1000 in 0.1% TBS-Tween-20 and 1% BSA and washed three times with the same buffer. Proteins were immunoprobed using an HRP-conjugated secondary antibody (dilution 1:3000) and chemiluminescent substrate (Clarity Western ECL substrate, Bio-Rad, Hercules, CA, USA). Blot image acquisition was performed using a Molecular Imager^®^ Gel Doc™ XR + system (Bio-Rad, CA, USA). The optical density of the protein bands was calculated after background subtraction and normalization to β-Actin using Image Studio 4.0 software (LI-COR Biotechnology).

### TUNEL assays

To further confirm the induced apoptosis, the TUNEL method was employed with an In situ Cell Death Detection Kit, Fluorescein 11684795910 (Roche, USA). Briefly, 5 × 10^4^ MDA-MB-231 cells were grown over glass coverslips into six-well cell culture clusters (Costar, USA) and incubated in the absence or presence of 700 μM rabeprazole or 70 μM auranofin for 24 h at 37 °C. Then, the cells were washed three times with PBS and incubated with Hanks balanced salt solution for 10 min at room temperature. Cells were fixed with 100% methanol at − 20 °C for 15 min; finally, the methanol was removed, and the cells were rehydrated by washing with PBS 2 times for 5 min each. Samples were stained with 1 mg/mL 4′,6-diamidino-2-phenylindole (DAPI), washed with PBS and incubated with proteinase K (20 mg/mL) for 30 min at room temperature. Then, the cells were washed with PBS and incubated for 30 min at room temperature with 0.3% H_2_O_2_ in methanol. Immediately after incubation with permeabilization solution (0.1% Triton X-100 in PBS) for 1 h at 4 °C, the cells were washed three times with PBS and incubated in the dark for 1 h at 37 °C with solutions A and B. Analysis was performed by fluorescence microscopy (Axiovert, Carl Zeiss. Germany).

### Caspase-1 assays

Because HsTIM accumulated in breast cancer cells but not in HMECs, we hypothesized that this condition is important to generate a favorable response to drug treatments. Thus, we performed assays to mimic HsTIM accumulation in normal cells by inhibiting caspase-1 as follows.

A stock solution (50 mM) of the selective caspase-1 inhibitor VX-765^62^, from BioVision (Milpitas, CA) was prepared in DMSO and diluted in HMEC medium to the appropriate concentrations. HMECs (5 × 10^6^) were incubated for 24 h as previously mentioned with or without 500 µM caspase-1 inhibitor. Then, 0.5 and 1.5 mM auranofin and rabeprazole, respectively, were added to the samples and incubated for another 24 h under the same conditions. At the end of incubation time, the cells were detached, washed and prepared for the following assays as previously described: nPAGE, cell viability assay, HsTIM activity assay, AGE quantification, assays to assess factors related to the induced cell death, and TUNEL assays. To corroborate the inhibition of caspase-1, caspase-1 activity was measured. In a buffer containing 100 mM HEPES (pH 7.2), 100 mM NaCl, 0.2% CHAPS, 20 mM EDTA, 10% glycerol and 10 mM DTT, 500 μg of total extract of cellular proteins, the reaction was initiated in a 96-well plate (total reaction volume was 100 µL/well) by the addition of 200 µM Ac-YVAD-pNA substrate.

The plate was then incubated for 4 h at 37 °C, and at the end of the incubation period, the absorbance at 405 nm was read using a microplate ELISA reader.

This assay is based on the ability of the active enzyme to cleave the chromophore pNA, as measured through determining the absorbance at 405 nm.

### In vivo experiments

Five- to seven-week-old female athymic Balb/c nude mice (Nu/Nu) were obtained from the Círculo ADN S.A. de C.V. and housed in the Unidad de Investigación Preclínica of the National Autonomous University of Mexico under specific pathogen-free (SPF) conditions with free access to autoclaved food and water; the mice were cared for following NIH guidelines for laboratory animals. For studies of the presence and effect of deamidated HsTIM in tumors, the group sizes were chosen based on previous experience, and mice were randomly assigned to one of the following experimental groups: nontreatment (*n* = 4), subcutaneously injected once with 20 × 10^6^ MDA-MB-231 cells and intraperitoneally administered sterile PBS three times a week; treated (*n* = 6), subcutaneously injected once with 20 × 10^6^ MDA-MB-231 cells and intraperitoneally administered 50 mg/kg rabeprazole three times a week; pretreated (*n* = 2), subcutaneously injected once with 20 × 10^6^ MDA-MB-231 cells previously incubated with 1 mM rabeprazole for 24 h. The viability of the cells was verified before injection, and the injection volume was adjusted to guarantee that at least 20 × 10^6^ of the injected cells were alive. Human cancer cells were implanted in mice by subcutaneous injection in the dorsal flank. The number of animals and all procedures followed protocols approved by the Institutional Animal Care and Use Committee for Care and Use (IACCUAC protocol UNIPREC-19-020).

Rabeprazole was intraperitoneal applied 7 days after the implantation of MDA-MB-231 cells. The animals were weighed, and the tumor length and width were measured using a digital caliper three times a week for 34 days. Using an established formula (0.52 × (length of the ‘long axis’ of the tumor) × (length of the ‘short axis’ of the tumor)^2^, tumor sizes were converted into tumor volumes^63^.

The experiment finished 24 h after the last drug application in each group, after which the animals were killed in a CO_2_ chamber. Tumors (when they were present) were dissected and prepared to analyze HsTIM as mentioned above.

The use of experimental animals in this study was approved by the Institutional Animal Care and Use Committee of National Autonomous University of Mexico, Faculty of Chemistry, Mexico (protocol code: UNIPREC-19-020).

## Supplementary Information

Supplementary Information.
