# Supplementary material for: Naturally occurring deamidated triosephosphate isomerase is a promising target for cell-selective therapy in cancer
Source: Sci Rep. 2022 Mar 7;12:4028. doi: 10.1038/s41598-022-08051-0 (PMC8901631; doi:10.1038/s41598-022-08051-0)
Supplement: Supplementary file 1 — Supplementary Information. [file 41598_2022_8051_MOESM1_ESM.pdf]

Naturally occurring deamidated triosephosphate isomerase is a promising target for cell-selective therapy in cancer.

Sergio Enríquez-Flores[1,*], Luis A. Flores- López[2], Ignacio De la Mora-De la Mora[1], Itzhel García-Torres[1], Isabel Gracia-Mora[3], Pedro Gutiérrez-Castrellón,[4] Cynthia Fernández-Lainez[5,7,8], Yoalli Martínez-Pérez[1], Alberto Olaya-Vargas[6], Paul de Vos[7], Gabriel López-Velázquez[1,*].

[1] Laboratorio de Biomoléculas y Salud Infantil, Instituto Nacional de Pediatría, CDMX, México; ignaciodelamora@yahoo.com.mx (I.D.-D.); itzheltorres@hotmail.com (I.G.-T.) yoalli89@gmail.com (Y.M.-P.)

[2] CONACYT-Instituto Nacional de Pediatría, Laboratorio de Biomoléculas y Salud Infantil, CDMX, México; luisbiolexp@gmail.com (L.F.-L.)

[3] Directora de la Unidad de Investigación Preclínica, Facultad de Química, Universidad Nacional Autónoma de México, CDMX, México; isabel.gracia@gmail.com (I.G.-M.)

[4] Hospital General Dr. Manuel Gea González, CDMX, México; inpcochrane@gmail.com (P.G.-C.)

[5] Laboratorio de Errores Innatos del Metabolismo y Tamiz, Instituto Nacional de Pediatría, CDMX, México; lainezcynthia@hotmail.com (C.F.-L.)

[6] Stem Cell Transplantation and Cellular Therapy, Instituto Nacional de Pediatría, CDMX, México; alberto.olaya@yahoo.com.mx (A.O.-V.)

[7] Department of Pathology and Medical Biology, University of Groningen, University Medical Center Groningen, Groningen, 9713 GZ, the Netherlands; p.de.vos@umcg.nl (P.d.V.)

[8] Posgrado en Ciencias Biológicas, Universidad Nacional Autónoma de México, CDMX, México

SUPPLEMENTARY MATERIAL.

Supplementary Table S1. Quantification of free Cys residues of the recombinant HsTIMs untreated or treated with the drugs.

HsTIM	Condition	Free Cys/subunit	Derivatized Cys /subunit**	Enzyme activity (%)
Non deamidated (WT)	Control*	4.85 ± 0.31	0	100
	+ Rabeprazole	3.82 ± 0.36	~ 1	95 + 6.7
	+ Auranofin	3.89 ± 0.37	~ 1	97 + 6.1
Deamidated (N16D)	Control*	4.91 ± 0.35	0	100
	+ Rabeprazole	1.12 ± 0.34	~ 4	1.4 + 0.7
	+ Auranofin	0.96 ± 0.42	~ 4	2.4 + 1.1

* Enzyme incubated in absence of drug.
** Obtained from the subtraction of the control (free Cys/subunit) and the corresponding drug (free Cys/subunit).

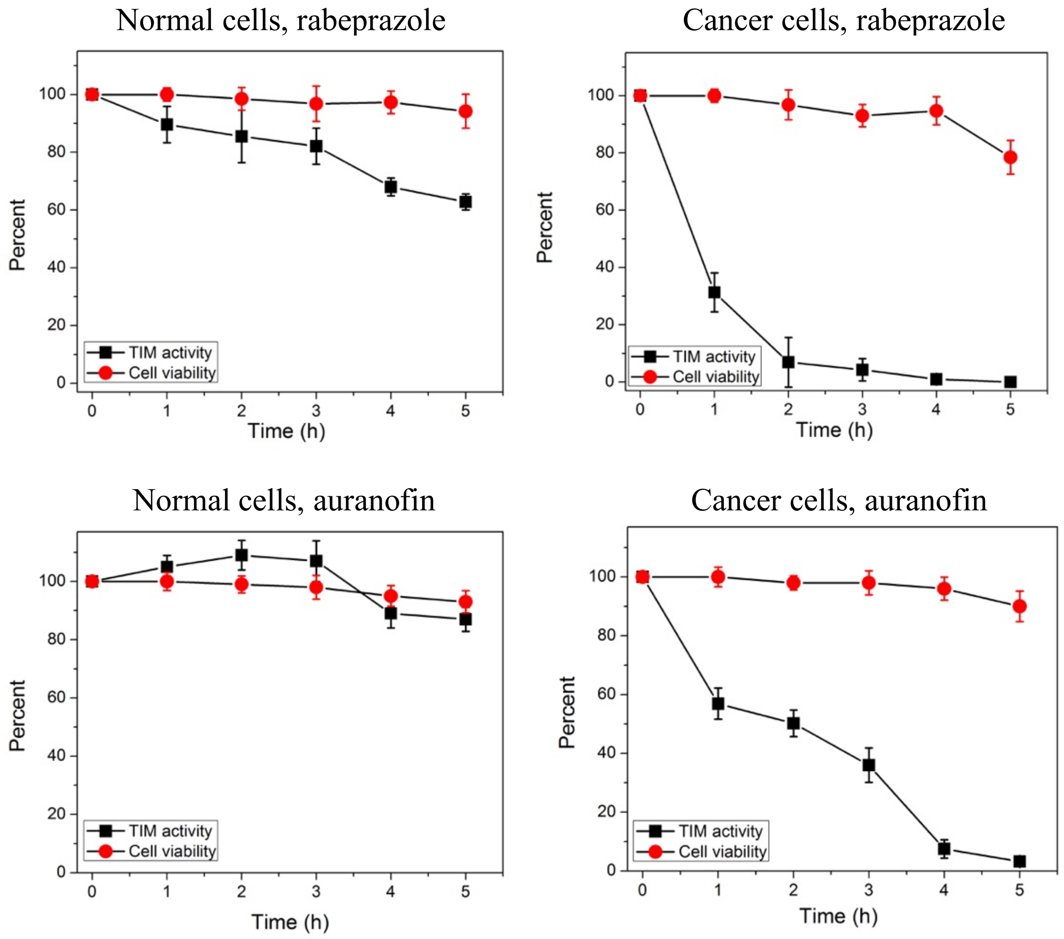

Supplementary Figure S1. Assays with high concentrations of drugs and short time intervals of incubation in HMECs and MDA-MB-231 cells. 1x10^5 cells/well were exposed to 5 mM of rabeprazole and 1 mM of auranofin, during 5 h with the culturing conditions mentioned in Material and Methods section. In each hour, cells were extensively washed, and their viability was determined with MTT, thereafter were lysed to measure the enzyme activity of HsTIM. Results are expressed as percent of residual enzyme activity, taken the values obtained in absence of the drugs as 100 %. The results are the mean of four independent experiments.

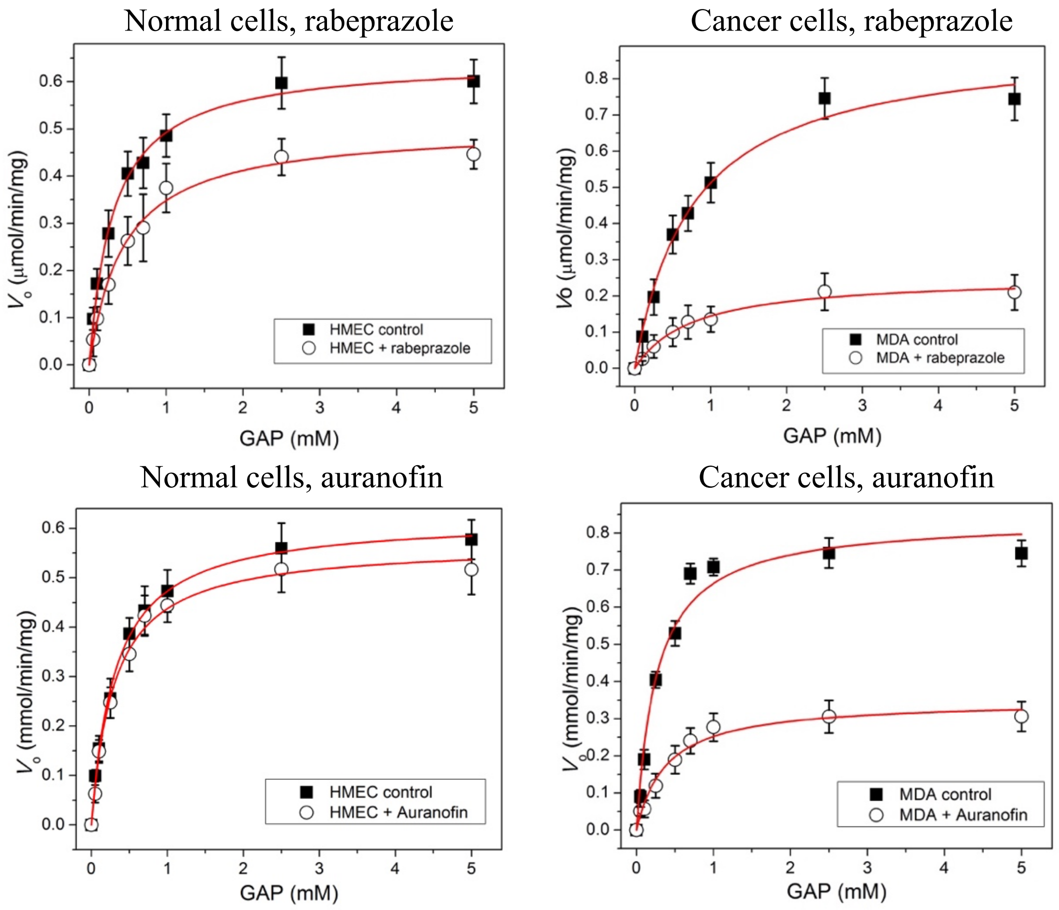

Supplementary Figure S2. Kinetic parameters of HMECs and MDA-MB-231 cells, untreated or treated with high concentrations of drugs and short time intervals of incubation. 1x10^5 cells/well were exposed to 5 mM of rabeprazole and 1 mM of auranofin, during 5 h with the culturing conditions mentioned in the Material and Methods section. In each hour, cells were extensively washed and lysed to measure the enzyme activity of HsTIM with increasing concentrations of the GAP substrate. Initial velocity data were measured in 100 mM triethanolamine, 10 mM EDTA pH 7.4, 0.2 mM NADH, 5 units of α-GDH, and increasing concentrations of GAP. The reaction was initiated by the addition of 100 µg/mL of cellular extract and the absorbance of NADH was measured spectrophotometrically at 340 nm. Lines are the fit to the Michaelis-Menten equation to adjust to kinetic parameters. The results are the mean of four independent experiments.

Supplementary Table S2. Kinetic parameters of cellular HsTIM in normal and cancer cells untreated or treated with the drugs.

**Cell type**	**Condition**	K_M **(mM)**	V_{max} **(µmol · min^{-1} · 5 X10^{5} cells)**
HMEC	Control	0.31 ± 0.01	0.62 ± 0.09
	+ Rabeprazole	0.44 ± 0.04	0.50 ± 0.04
	+ Auranofin	0.29 ± 0.02	0.56 ± 0.03
MDA-MB-231	Control	0.29 ± 0.04	0.83 ± 0.03
	+ Rabeprazole	0.5 ± 0.10	0.25 ± 0.02
	+ Auranofin	0.48 ± 0.06	0.30 ± 0.05

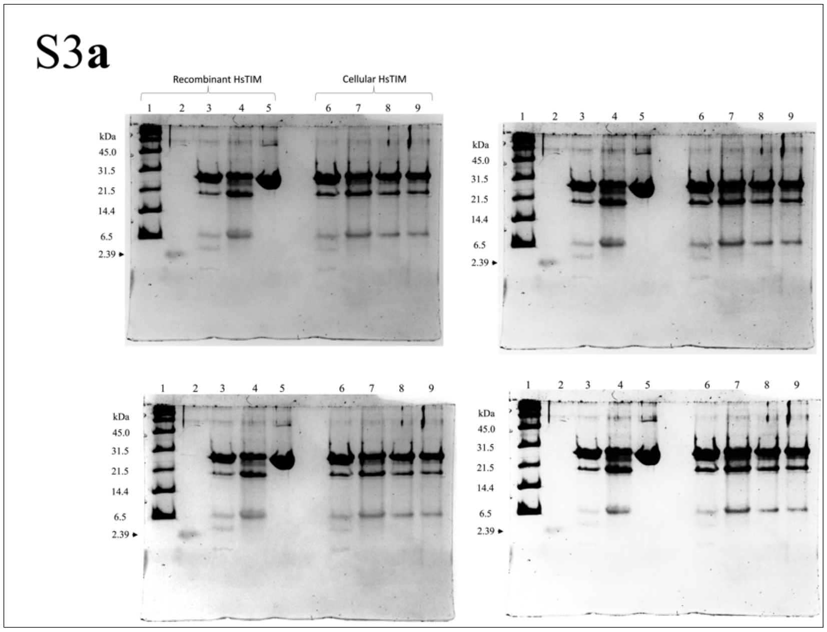

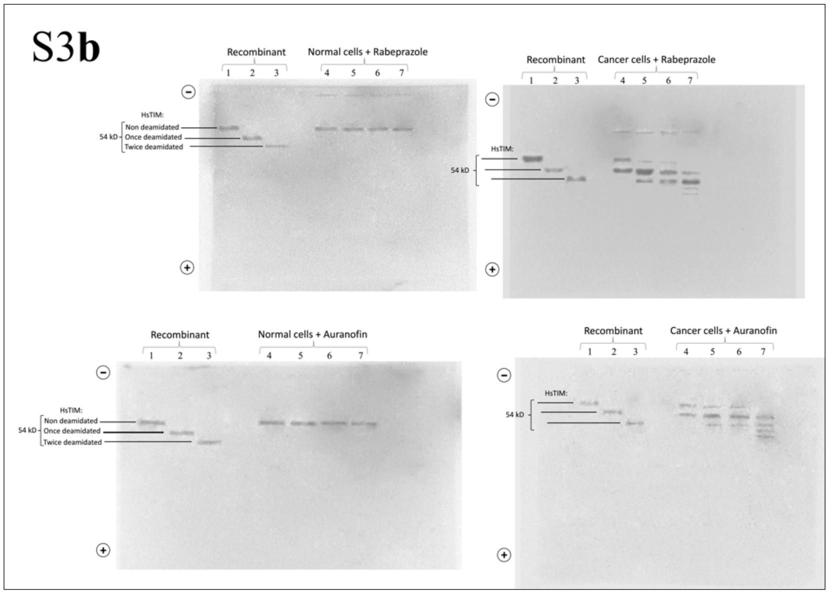

Supplementary Figure S3. Full-length gel and blots of Fig. 2**a**. Panel S3**a** corresponds to the same gel with different contrast exposures. Panel S3**b** corresponds to the blots from nPAGE of recombinant and cellular HsTIMs; the arrows indicate the acidic species of HsTIMs.

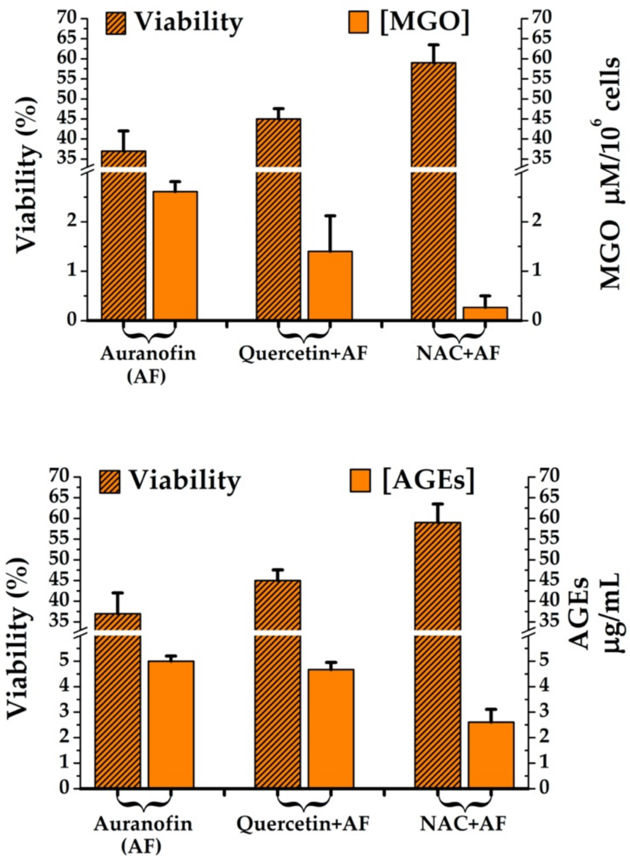

Supplementary Figure S4. Effects of quercetin and N-acetylcysteine in cancer cells treated with auranofin. **a**, viability and production of MGO in presence of a quencher of ROS (quercetin) or a scavenger of MGO (NAC). **b**, viability, and production of AGEs in presence of a quencher of ROS (quercetin) or a scavenger of MGO (NAC).

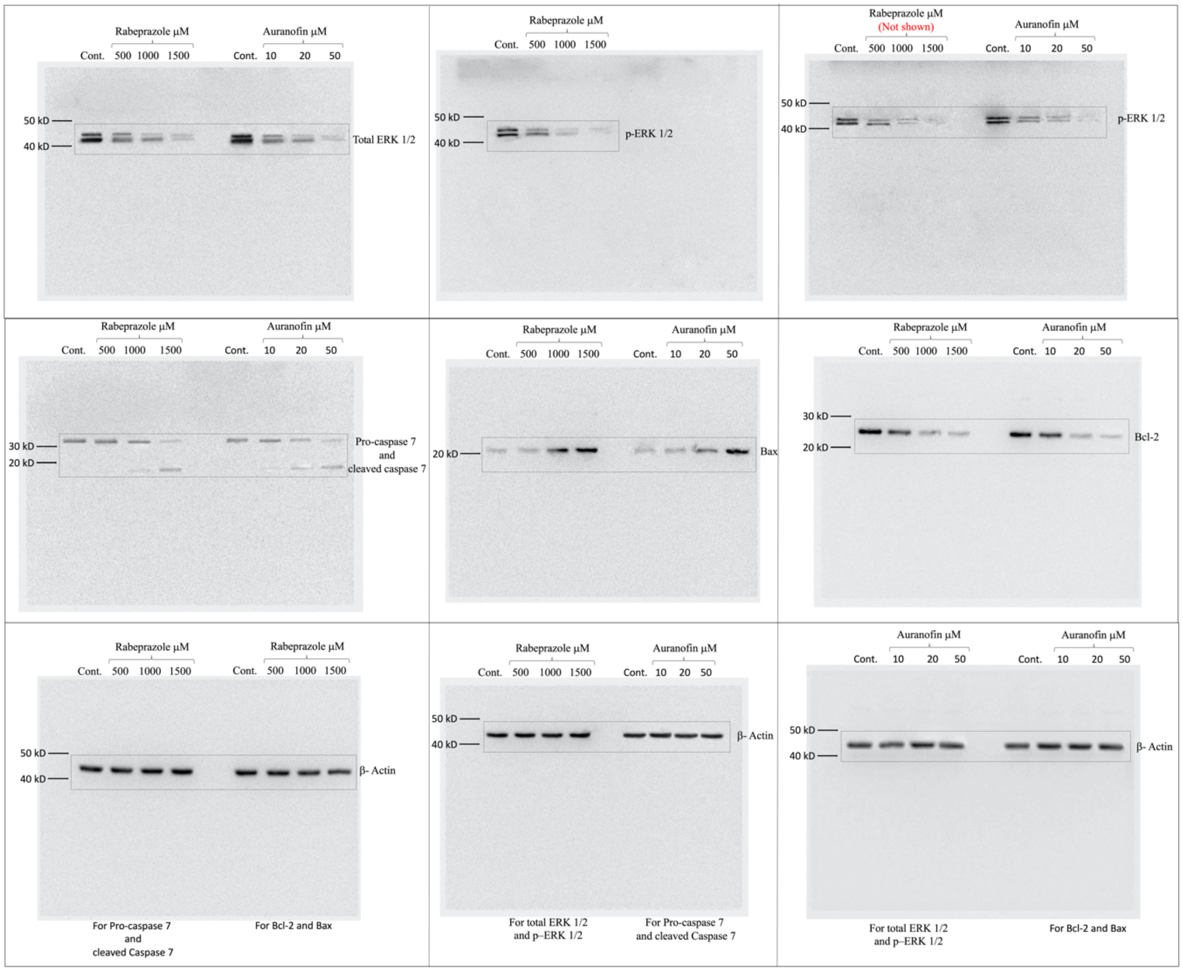

Supplementary Figure S5. Full-length blots corresponding to Figures 4**a,** 4**b**, and 4**c**.

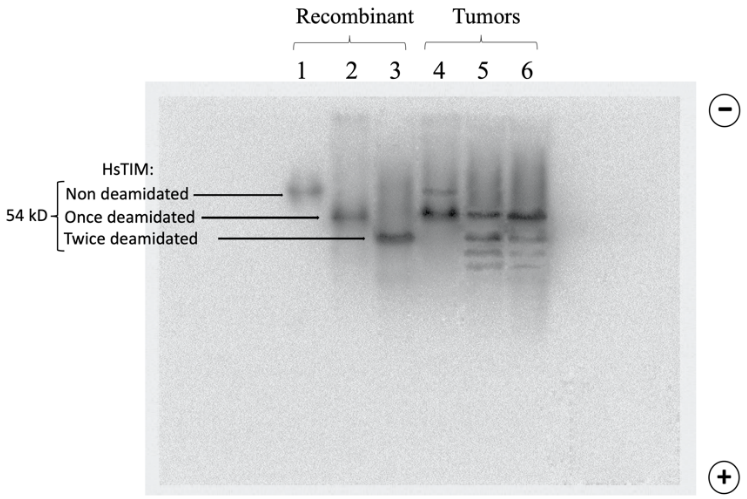

Supplementary Figure S6. Full-length blot corresponding to Fig. 5**c**. The arrows indicate the acidic species of HsTIMs and the circles indicate the polarity of gel.

Supplementary Table S3. Cell viability and enzymatic activities of caspase-1 and HsTIM in normal cells untreated or treated with the drugs.

**Normal cells (HMEC)**	**Cell viability (%)**	**Caspase-1 activity (%)**	**HsTIM activity (%)**
Control (DMSO)	100	100	100
Caspase-1 inhibitor	89.4 ± 4.3	23 ± 6.3	145.5
+ Rabeprazole	91.7 ± 6.2	85.2 ± 7.8	71.2
Caspase-1 inhibitor + Rabeprazole	59.3 ± 5.8	7.6 ± 3.4	64.6
+ Auranofin	92.1 ± 4.8	89.6 ± 5.3	84.7
Caspase-1 inhibitor + Auranofin	55.9 ± 6.7	4.6 ± 3.4	67.8

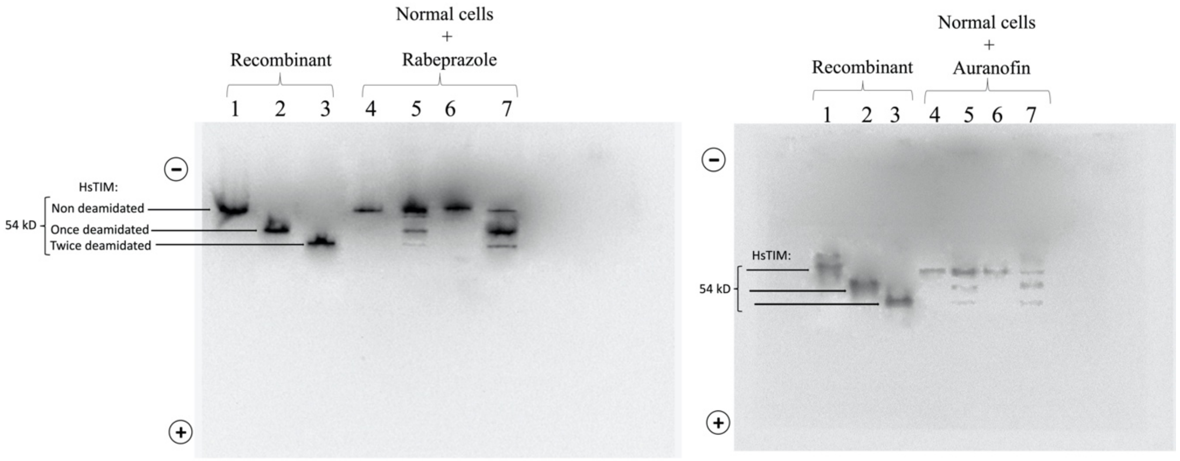

Supplementary Figure S7. Full-length blots corresponding to Fig. 6**a**. The arrows indicate the acidic species of HsTIMs and the circles indicate the polarity of gel.

Supplementary Table S4. Enzyme activity of HsTIM from tumors of the xenograft rodent model.

Tumor from experimental groups	HsTIM activity (%)
Untreated	100
Treated	46.1 ± 2.3
